# Supplementary material for: Quantifying nursing care delivered in Kenyan newborn units: protocol for a cross-sectional direct observational study
Source: BMJ Open. 2018 Jul 23;8(7):e022020. doi: 10.1136/bmjopen-2018-022020 (PMC6059345; doi:10.1136/bmjopen-2018-022020)
Supplement: Supplementary data [file bmjopen-2018-022020supp001.pdf]

Quantifying neonatal nursing care observational checklist

Hospital logistics

1. Start of observation: Date ☐ Time ☐
2. End of observation: Date ☐ Time ☐
3. Shift: Weekday day ☐ Weekday night ☐

   Weekend day ☐ Weekend night ☐
4. Total number of patients in the ward: ☐
5. Total Number of: Admissions ☐
   - Discharges ☐
   - Referrals ☐
   - Death ☐
6. Number of nurses on shift: Straight day shift ☐
   - Morning shift ☐
   - Afternoon shift ☐
   - Night shift ☐
7. Number of other staff providing care other than the nurse:
   - Doctor ☐
   - Clinician ☐
   - Health care assistant ☐
   - Student nurse ☐
   - Student CO ☐
   - Upgrading nurse students ☐

   Others ________________________________

Bio data

8. Child category: A. (Critical/HDU) ☐

   B. (Acute) ☐

   C. (Stable) ☐

9. Date of admission ☐

10. Age (days/ If day 1 of life give hours) ☐

11. Gender: Male ☐

    Female ☐

12. Admission diagnosis: ____

    ____

13. Current diagnosis: ____

    ____

14. Birth weight (grams) ☐

15. Current weight (grams) ☐ Date taken ☐

16. Type of delivery: SVD ☐ CS ☐ AVD ☐

Tasks	Frequency of task	Expected number tasks to be done(12hours/shift)	Tasks_done				Time task done				Comments
**Routine Tasks**											
**Handing over**		1									
**Patient assessments performed each shift**		1									
**Cleaning of baby**		1									
**Change of linen**		1									
**Checking incubator settings**		1									
**Nurse attends ward round with doctor(s)**		1									
**Weight check**		1									
**Elimination care**		1									
**Discharge planning**		1									
**Communication/counselling to parent**		1									
**Hand washing/ Hand rub using sanitizer**		1									
**Cord care**		1									
**Checking cannula site**		1									
**Regular Tasks**											
**Checking vital signs***	4/6 Hourly	3									
**Temperature check***	4/6 Hourly	3									
**Pulse check***	4/6 Hourly	3									
**Respiratory rate check***	4/6 Hourly	3									
**SpO2 check***	4/6 Hourly	3									
**Turning**	4 Hourly	3									
**Cup/spoon feeding**	3Hourly	4									
**Breast feeding**	3 Hourly	4									

*Dependend on neonate category

Tasks	Frequency of task	Expected number tasks to be done(12hours/shift)	Tasks_done	Time task done	Comments
**Critical Tasks**					
**Naso gastric tube feeding**					
**Checking for correct position of tube**	3 Hourly	4			
**Checking gastric aspirate before feeding**	3 Hourly	4			
**Checking actual volume of feeds**	3 Hourly	4			
**Actual feeding**	3 Hourly	4			
**Positioning baby after feeding**	4 Hourly	4			
**Phototherapy**					
**Turning/positioning**	4 Hourly	3			
**Skin assessment**	6 Hourly	2			
**Checking eyes if protected from damage**	6 hourly	2			
**Changing eye pad**	12 Hourly	1			
**Eye care**	12 Hourly	1			
**Oxygen therapy**					
**Checking tube position and nostril-care, damage**	3 Hourly	4			
**Regulating oxygen flow**	12 Hourly	1			
**Intravenous drug administration**					
**Dilutions and checking compatibility**		2			
**Review of treatment sheet**		2			
**Flushing cannula before administering drug**		2			
**Administration of medication**		2			
**Flushing cannula after giving medication**		2			

Tasks	Frequency of task	Expected number tasks to be done(12hours/shift)	Tasks_done	Time task done	Comments
**Intravenous Fluid administration**					
Review of treatment sheet		1	☐	☐	
Priming of giving set		1	☐	☐	
Flushing cannula before starting the fluid		1	☐	☐	
Administration and regulating flow rate		1	☐	☐	
**Kangaroo Mother Care**					
Support for KMC		1	☐	☐	
Supervision of mother during KMC		1	☐	☐	

Documentation of tasks

Tasks	Done	Not done	Comments
Neonatal assessment (nursing cardex)	☐	☐	
Planned care (cardex/care plan)	☐	☐	
Vital signs (observation charts/cardex)	☐	☐	
Treatment (treament sheets)	☐	☐	
Ward round comments (cardex)	☐	☐	
Phototherapy (cardex)	☐	☐	
Feeds (feeding chart)	☐	☐	
Oxygen therapy (cardex)	☐	☐	
Health talks/ Communication to parent (cardex)	☐	☐	
Fluids (input output chart)	☐	☐	

**Turning/position(cardex and/or turning chart)**	☐	☐	
**Alternate weight check**	☐	☐	

Treatment

**Drug name**	**Frequency**	**Times administered in the last 24 hours**	**Comments**

Tasks observed for other babies

**Task**	**Observed**	**Comments**
**Administration of vaccines**		
**Taking venous blood**		
**Taking heel prick**		
**Blood/exchange transfusion**		
**CPAP(setup of machine n tubings)**		
**Resuscitation**		

Other tasks observed not mentioned above

