# Supplementary material for: Quantifying nursing care delivered in Kenyan newborn units: protocol for a cross-sectional direct observational study
Source: BMJ Open. 2018 Jul 23;8(7):e022020. doi: 10.1136/bmjopen-2018-022020 (PMC6059345; doi:10.1136/bmjopen-2018-022020)
Supplement: Author's manuscript [file bmjopen-2018-022020.draft_revisions.pdf]

BMJ Open is committed to open peer review. As part of this commitment we make the peer review history of every article we publish publicly available.

When an article is published we post the peer reviewers' comments and the authors' responses online. We also post the versions of the paper that were used during peer review. These are the versions that the peer review comments apply to.

The versions of the paper that follow are the versions that were submitted during the peer review process. They are not the versions of record or the final published versions. They should not be cited or distributed as the published version of this manuscript.

BMJ Open is an open access journal and the full, final, typeset and author-corrected version of record of the manuscript is available on our site with no access controls, subscription charges or pay-per-view fees (http://bmjopen.bmj.com).

If you have any questions on BMJ Open's open peer review process please email info.bmjopen@bmj.com

BMJ Open

Quantifying nursing care delivered in newborn units: Protocol for a direct-observational study

Journal:	*BMJ Open*
Manuscript ID	bmjopen-2018-022020
Article Type:	Protocol
Date Submitted by the Author:	02-Feb-2018

Complete List of Authors:	Gathara, David; Kenya Medical Research Institute/Wellcome Trust Research Programme, Department of Public Health Research<br>Serem, George; Kenya Medical Research Institute/Wellcome Trust Research Programme, Department of Public Health Research<br>Murphy, Georgina ; Kenya Medical Research Institute/Wellcome Trust Research Programme, Department of Public Health Research; Bill and Melinda Gates Foundation<br>Abuya, Nancy; Kenya Medical Research Institute/Wellcome Trust Research Programme, Department of Public Health Research; Nairobi City County, Department of Curative and Preventative Services<br>Kuria, Rose; Kenya Medical Training College, Department of Nursing<br>Tallam, Edna; Nursing Council of Kenya, Department of Registration and Licensing<br>English, Mike; University of Oxford, Nuffield Department of Medicine and Department of Paediatrics; Kenya Medical Research Institute/Wellcome Trust Research Programme, Department of Public Health Research

Keywords:	Health & safety < HEALTH SERVICES ADMINISTRATION & MANAGEMENT, NEONATOLOGY, STATISTICS & RESEARCH METHODS

SCHOLARONE™
Manuscripts

Quantifying nursing care delivered in newborn units: Protocol for a direct-observational study

Investigators and Institutional Affiliations

David Gathara[1*], George Serem[1], Georgina A. V. Murphy [1, 2], Nancy Abuya[1,3], Rose Kuria[4], Edna Tallam[5], Mike English [1,2]

Affiliations

1. Department of Public Health Research, Kenya Medical Research Institute/Wellcome Trust Research Programme, Nairobi, Kenya
2. Nuffield Department of Medicine and Department of Paediatrics, University of Oxford, UK
3. Department of Curative and Preventative services, Nairobi City County, Kenya
4. Department of Nursing, Kenya Medical Training College, Kenya
5. Department of Registration and Licensing, Nursing Council of Kenya

*Corresponding author
Dr David Gathara
P.O Box 43640 00100
Kenya Medical Research Institute/Wellcome Trust Research Programme
Nairobi, Kenya
Email: Dgathara@kemri-wellcome.org
Phone: (+254) 721659321; (+254) 730 162 000

Abstract

Introduction: In many African countries, including Kenya, a major barrier to achieving child survival goals is the slow decline in neonatal mortality that now represents 45% of the under-5-mortality. Improved facility based care for sick or preterm newborns is central to efforts to improve newborn outcomes. In newborn care, nurses are the primary care givers and are essential in the delivery of safe and effective care. However, due to high patient workloads and limited resources, nurses may often consciously or unconsciously have to prioritise the tasks and the care they provide resulting in some tasks being left undone or partially done (missed care). Missed care has been associated with poor patient outcomes in high income countries. However, missed care, examined by direct observation, has not previously been the subject of research.

Methods and analysis: The aim of this study is to quantify the nursing care provided to newborns within newborn units. We will undertake a cross-sectional study utilising direct observational methods within newborn units in 6 health facilities in Nairobi City County across the public, private-for-profit and private-not-for-profit sectors. We will report the overall prevalence of care left undone, the common tasks that are left undone and describe any sharing of tasks with people not formally qualified to provide care that may represent coping strategies. In a secondary analysis, will will explore variations in care

For peer review only - http://bmjopen.bmj.com/site/about/guidelines.xhtml

done (or left undone) by sector, nursing working-shift, nurse-patient ratios and disease severity of the baby.

Ethics and dissemination: Ethical approval for this study has been granted by the Kenya Medical Research Institute (KEMRI) Scientific and Ethics Review Unit (KEMRI/SERU/CGMR-C/065/3404). Findings from this work will be shared with the participating hospitals, an expert advisory group that comprises of members involved in policy making and more widely to the international community through peer-reviewed journals and conferences.

Strengths and limitations

- New approaches to quantifying nursing care delivered or left undone. The use of direct observational methods that have not been previously used in LMICs. Most nursing research on quantifying care delivered is based on nurse- or patient- reported surveys.
- The different sectors (public, private-for-profit, private-not-for-profit) in health service provision have included in this study. However, our scope is limited by the few number of newborn to be observed within each hospital
- Highlighting the extent and magnitude of care left undone will provide important insights on the quality of care available to sick newborns, highlight human resource issues warranting attention and will likely influence recommendations on staffing norms and how care is organized and delivered in newborn units.

Introduction

Despite progress globally, most African countries including Kenya have made insufficient progress in reducing child mortality. In most countries, this can be partly attributed to only slow declines in neonatal mortality. As a consequence about 45% of child mortality is neonatal mortality[1]. Of these neonatal deaths, approximately 75% occur in the first 7 days of life and half of these within 24 hours of life[2][3]. A recent review by Bhutta and colleagues indicated that high impact low cost interventions could avert more than 71% of neonatal deaths with 82% of this effect being attributable to facility-based care[4]. However, reports from low income settings highlight that the quality of newborn care in health facilities is often poor[5][6][7]. Therefore, strengthening the quality of facility based care for newborns will be essential in improving newborn outcomes.

Human Resources for Health (HRH) inadequacies are major factors limiting neonatal service delivery quality [8]. Globally, the shortage of health workers is estimated currently at over 7 million and by 2035 the deficit is estimated to be 12.9 million[9]. The shortages in the available workforce are worst in developing countries where inequitable distribution of available health workers may compound the problems. In Kenya, Wakaba and colleagues reported that public sector nursing densities ranged between 1.2 to 0.008 per 1000 population across counties[10] compared to an internationally suggested

For peer review only - http://bmjopen.bmj.com/site/about/guidelines.xhtml

minimum health workforce threshold of 2.5/1000 population for doctors, nurses and midwives. In Nairobi County, the nurse densities ranged between 0.21 to 0.40 per 1000 population[10].

There is little specific exploration of the impact of nursing workforce shortfalls on inpatient care in Low and Middle-Income Countries (LMIC). Yet to improve quality of care it is essential that we understand who delivers care (what tasks are done by whom), how is care delivered (how are tasks performed) and critically what tasks are left undone. In most health systems, nurses are gatekeepers of the health care being delivered. They are vested with the responsibility of delivering interventions prescribed by other providers (doctors, nutritionists etc) in addition to providing nurse initiated interventions[11]. As a consequence, few interventions reach the patient without the involvement of the nurse. Yet the few existing evaluations of the quality of care provided to newborns in LMIC have focused on the more medical aspects of care[6,12,13][14]. In LMIC facilities, large patient workloads, insufficient staff and resources, urgent patient situations, and unexpected rises in patient volume and/or acuity on the unit (among other factors) might result in all facets of nursing care being delayed or neglected. This phenomenon has been described as 'implicit rationing'[15], 'missed care'[16], or 'unmet nursing care needs'[17], 'care left undone'[18], or 'task incompletion'[19]. Hereafter, we use the term missed care to encompass all of these terms. Such missed care may have a particularly devastating impact on outcomes in newborn units where nurses are the primary care givers to this highly dependent group.

Justifying a focus on missed nursing care several studies have reported associations between missed care and patient outcomes[20,21][22]. Although there is a growing literature on missed care the majority is from high resource settings, with only one study in South Africa[23] providing an early formal attempt to quantify the extent of the problem in this middle income country. Furthermore, almost all the literature on missed care is based on nurse surveys with only two focusing on newborn care provision[24,25]. Although, nurse surveys on missed care have proven useful, there is a call to undertake more research with a special focus on objective observational methods as no studies of this type were identified in a recent systematic review [22]. The proposed study aims to characterise the care delivered (tasks done or left undone and who does these tasks) to newborns receiving care within newborn units in Nairobi, Kenya by making direct observations of care being provided. This will provide in-depth and objective insights on missed care with particular reference to locally agreed standards for nurses providing neonatal care.

For peer review only

Methods and analysis

This is a cross-sectional study that will utilise direct observation of care provided to individual newborns with the aims of describing the nursing care given and missed within newborn units in 6 hospitals.

Study site (geographical)

The proposed research work will be undertaken as part of a set of work being conducted in collaboration with Nairobi City County. This collaboration includes work to characterise all the facilities providing 24/7 inpatient newborn care in Nairobi [26] and ethnographic work to understand the wider context and practice of neonatal nursing. Nairobi county has 34 health facilities providing 24/7 inpatient newborn care, of these 2 health facilities declined to take part in prior quality of care surveys[27].

For peer review only - http://bmjopen.bmj.com/site/about/guidelines.xhtml

Excluding the military health facility with restrictive admission policy, the remaining 31 health facilities that form the population for this study provide 99% of all inpatient neonatal care.

This current study will focus on primary referral (secondary care) facilities that have more than 100 neonatal admissions annually. Thirteen facilities meet these criteria and together provide care to over 96% of the sick-newborn population accessing care within Nairobi County. These 13 facilities will be stratified by workload (newborn admissions per year <=500 low; >500 high) and six health facilities purposefully selected to ensure representation of 2 hospitals in each of the public, private-not-for profit and private-for-profit sectors, with one high and one low workload facility in each sector. Purposeful selection will be used as it is important in this sensitive and innovative work to have the strong support of the hospital administration. This initial work will therefore help illustrate the nature and magnitude of the challenge of missed care but not make claims to provide a statistically representative picture of missed care which would be challenging given the great diversity of facilities found in Nairobi, some with as few as 10 neonatal admissions per year[27].

Study populations

All newborns admitted within a newborn unit in the 6 selected health facilities over the period of the study in each facility will form the potential study population. However, newborns meeting the following exclusion criteria will not be observed: i) newborns requiring specialised treatment to whom the draft minimum nursing standards for neonatal care[28] may not be applicable, for instance newborns with gross malformations or those receiving post-operative care, ii) newborns who are critically ill and at risk of death within a 12-hour observation period as defined by the clinician in charge of the newborn unit for whom observation might cause distress to families, iii) newborns for whom guardians do not provide consent, and iv) newborns receiving care from nurses or guardians who decline to be observed in the care provision process.

Sampling procedures

To describe the care being provided to sick newborns across the spectrum of inpatient services they receive, we are aiming to sample time in 12 hour shifts randomly using the steps described in figure 1.

Figure 1: Steps for the sampling procedure

Step 1

Care within the NBU can be organised in a way that babies requiring different levels of care are in different sections/rooms. Intensive care employing invasive mechanical ventilation is not available outside tertiary hospitals in Kenya and thus definitions for different levels of newborn care provided by County Hospitals were adopted. These are taken from draft nursing standards for neonatal care that categorise NBU sections (and thus babies) in the following way[28]. **Category A (high dependency unit, HDU)**: Babies on oxygen/CPAP and IV fluids who are often acutely ill and unstable and require the closest monitoring. **Category B**: Babies who have stabilized but may still be ill and receiving for example assisted feeding (NG feeds), IV drugs; or being observed for convulsions or apnoea. **Category C**: Babies who are quite stable who should be receiving KMC, or stable abandoned babies, or recovering babies

For peer review only - http://bmjopen.bmj.com/site/about/guidelines.xhtml

requiring completion of treatment such as the last doses of antibiotics or transitioning to oral feeding. Our primary sampling strategy will be based on identifying these organisational sub-sections (Category A; B; C rooms or incubator/cot spaces) in each facility. Where facilities have no clear organisational demarcation into Category A, B and C sub-sections we will adapt the observations to suit the organisation of care in each facility. However, we will endeavour to identify and select newborns with varying degrees of illness severity who would be classified as meeting criteria for Category A, B or C care for observation in such settings.

Step 2

In each hospital, a random sample of twelve shifts/time blocks of 12 hours (144 observation hours per hospital) will be selected from within a 3 week period stratified by both weekdays and weekends as well as night and day shifts as care has been shown to vary across these periods[29,30]. The 12 shifts will be divided equally across the NBU sections within a hospital where there is >1 section. Where all babies are cared for in one room without clear sub-sections all observation periods will be conducted in this same setting with efforts to observe babies requiring different levels of care.

Step 3

We feel that it is logistically feasible for one observer to make direct observations of 3 babies located in adjacent cots in the same ward area at one time. Therefore, for each shift and section, 3 babies who meet the inclusion criteria will be purposefully selected to ensure babies are in one ward area and within close proximity to allow direct observations. In smaller units where the number of babies per section may be less than 3, we will observe all the babies available in the section and classify the baby as eligible for care in category A, B or C.

Sample size

This is an exploratory cross-sectional study and as such, we illustrate the precision with which we can report proportions of tasks done (or not done) assuming different sized denominators. The size of the denominator is related to the recommended frequency that tasks should be performed (see figure 2). To estimate the precision of reporting we have used a sample estimation approach for cluster designs and assuming a design effect of 2 to adjust for clustering of observed tasks within newborns within hospitals.

At most we expect to recruit 216 newborns across 6 hospitals (36 newborns per hospital with 3 babies for each of the 12 shifts/time blocks). For tasks that would be conducted with an expected frequency of once in 12 hours (eg. IV pencillin administration) we might therefore observe 216 task opportunities. Taking a (statistically) conservative assumption that on 50% of expected occasions the task is observed to be done then we could report this with a precision ± 13.4%. Similarly, if a task should be conducted every 6 hours then the denominator would be 432 expected tasks and if half were observed the precision of this estimate would be ± 6.7%.

Not all babies observed will require all tasks. For example, some babies may not be receiving intravenous drugs. This will reduce the effective size of the denominator and reduce the precision we

For peer review only - http://bmjopen.bmj.com/site/about/guidelines.xhtml

can report. Similarly, if babies have to leave the area of observation (for example they are moved to a new ward area or are sent for X-ray etc) this may reduce the number of expected tasks that can be observed. In addition, failure to recruit 3 babies at each 12-hour shift at each facility may also reduce the number of tasks observed. We illustrate the effect this has on the precision of reported estimates in figure 2, which illustrates that with as few as 30 expected tasks reporting a precision of ± 20.5% is possible.

As this work is a first of its kind (globally as far as we are aware) we feel that providing estimates of the proportion of tasks done/left undone with a precision of approximately ± 20% will be sufficient to provide valuable insights into the challenges faced by nurses in providing newborn care.

Figure 2: Precision levels for different newborn sub-populations and tasks

Procedures

For each newborn being observed, the diagnosis and disease severity information will be collected from medical records. At the beginning of every shift for which observations will be made, the total number of nursing tasks expected to be delivered will be determined with reference to the medical and nursing records (for specific interventions) and general aspects of care each baby should receive. For each of the newborns selected to participate in the study, we will make direct observations on how often certain routine nursing tasks (listed in table 1) are undertaken in a 12-hour shift (7am -7pm) using an observation checklist. Observations will be stopped if a baby is transferred out of a section or discharged, however, we will use the data collected up to the point of exit and the number of observation hours will be documented. If the babies' condition changes and their category changes but they remain in the same observation area, we will document this change and revise the expected number of tasks.

The observer will be stationed in a ward area where they do not obstruct care provision but can observe the care being provided to the newborns selected for observation. Because most of the documentation activities happen at the nurses' desk/station, all tasks related to documentation cannot be observed from the cot-side. The documentation tasks for the babies under observation will be evaluated at the end of each 12-hour shift for which observations were made. We acknowledge that 12 hours is a long period and the observer's efficiency for making observations might reduce as time progresses within an observation time block and study period. We will factor in rest periods in the 12-hour shifts that coincide with when nurses take their breaks for instance, during tea and lunch breaks. Further, to allow enough rest between observation time blocks, we will aim to have a maximum of 3, 12-hour observation periods per week per observer.

The observation checklist is based on nursing standards produced after two expert group meetings held in November 2015 and July 2016 at the KEMRI Wellcome Trust Research Programme offices. The nurse stakeholder group comprises of expert nurses who are in the teaching profession or senior practitioners and including the acting Chief Nursing Officer for Kenya. They defined a minimum standard for performing nursing tasks on newborn units[28]. A sub-set of tasks were identified as critical (listed in table 1) by the nurse stakeholder group that should be performed by nurses and not by other personnel

For peer review only - http://bmjopen.bmj.com/site/about/guidelines.xhtml

due to the skills required when they are delivered. Where necessary tasks are broken down into manageable observable task components to facilitate observation. For instance, naso-gastric feeding is broken down into insertion of the NGT (as required), checking for gastric aspirate before feeding, counter checking feed volumes to be given with prescription, actual feeding and charting the feeds given. The purpose of observation is not to assess how well any particular aspect of a task is done (eg. the care taken in administering naso-gastric feeds) but simply to determine if the task (or task component) was or was not done at all.

Prior to the start of the study, the observation checklist will be extensively piloted over a period of 6 weeks to determine the quantity and quality of data that can be reasonably gathered and will be adapted as needed.

Table 1: Routine and critical tasks for observation

Routine tasks	Critical tasks
Patient assessment at the beginning of each shift<br>Cleaning of the baby<br>Changing baby's linen<br>Changing the baby's position<br>Checking incubator settings<br>Attendance at ward round and active note taking<br>Weighing<br>Elimination care<br>Communication/counselling parents<br>Cord care<br>Vital signs<br>• Pulse rate<br>• Temperature<br>• Respiratory rate<br>• Oxygen saturation<br>Documentation<br>• Updating the nursing cardex<br>• Discharge and admission registration	Nasal gastric feeding<br>• Insertion of the NGT<br>• Testing whether it is in the correct position<br>• Checking for gastric aspirate before feeding<br>• Preparation of feeds and counter checking feed volumes<br>• Actual feeding and charting the feeds<br>Intravenous drug/fluid administration<br>• Review treatment sheet<br>• Checking cannula sites – care<br>• Regulating flow<br>• Input/output charting for fluids<br>• Document treatments given<br>Oxygen therapy<br>• Fixing of oxygen/nasal prongs<br>• Checking tube position and nostril –care, damage<br>• Initiating and regulating oxygen flow<br>• Documenting oxygen treatment<br>Photo therapy<br>• Baby positioning<br>• Eye pad in place<br>• Checking eyes for damage<br>• Check and monitor phototherapy settings<br>• Documentation of phototherapy<br>Support for KMC

For purposes of this study, we adopted the definition of missed care reported in wider literature[22,31], and was therefore defined as care that the nursing advisory group regard as necessary (primarily nursing tasks) as part of routine newborn care that are left undone or are

For peer review only

For peer review only - http://bmjopen.bmj.com/site/about/guidelines.xhtml

delivered by any person other than the nurse and who is not under direct observation/supervision by the nurse. For example, tasks done by a senior student nurse who is being supervised to conduct nasogastric feeding by a qualified nurse in the room at the time and focused on the supervision will be regarded as done. If the same student does the task while the nurse supervising is in a different part of the ward it will be regarded as not done (with a record of who completed the task made). Additional data to be collected related to each block of observation time will include data on the nurse patient ratio for the ward as a whole, what additional staff are present within the unit e.g nursing students, support staff etc and patient workloads within the different sections of the ward.

Practical considerations to undertake direct observations

Observations will be made by a person familiar with the hospital environment, equipment, processes (like ward rounds) and language but who is not a nurse or clinician (doctor/clinical officer). Given the potential sensitivity of this form of observation it is important that this person is considered a professional rather than an outsider (who might not be bound by professional codes of confidentiality). Having an observer who has an understanding of the setting but who is not a clinician or nurse may also overcome problems of the observer making judgements about what is being observed based on their own standards of practice or being influenced as they make observations by professional allegiances (eg. a nurse observer may not wish to record that a task is left undone). There might also be ethical challenges for an observer with a clinical/nurse background who might feel obliged to intervene in the provision of care or become co-opted to complete tasks. An observer who is not licensed to offer the form of interventions/care being given to babies will still however be able to report to health care providers within the unit in the event they identify gaps or situations in which newborns are put at risk. For instance, if they saw a newborn was convulsing or vomiting they would alert the nurse. Suitable backgrounds for observers might include nutritionists, health records officers, ward based clerical workers, or laboratory or pharmacy staff with at least two-year's experience within hospital settings. In this study nutritionists will be recruited to undertake the observations.

We will recruit six observers who meet the above criterion. The observers will be trained for a period of 2 weeks by one of the study personnel who has a nursing background and has 2 years' experience providing care to newborns within a newborn unit. Standard operating procedures will be developed and will serve as a guide for the observational work. During the training period, the observers and the trainer will do observations and comparisons will be made. Any differences will be discussed and where necessary the SOPs will be revised to improve on clarity.

To reduce the Hawthorne effect where nurses might change the way they provide care when they are being observed, the observer will spend at least 1 week in the facility before starting the observational work. This will also allow him/her to familiarise themselves with the environment, to explain the study to staff and parents and for the staff within the NBU to get used to them. In addition, the observer will make it clear that the observations are not an assessment but a means of understanding what care it is possible to provide given existing resource constraints. Formal study observations will then begin in the period after the one-week familiarisation period. This approach is supported by evidence that health

For peer review only - http://bmjopen.bmj.com/site/about/guidelines.xhtml

care providers change their practice slightly when the observations start but these changes are short-lived and quickly dissipate with health care providers soon reverting to their previous practice[32,33].

Data Management and analysis

Data will be collected on paper-based observation checklists, one for each baby that is the focus of observation. No participant identifiable data will be collected and data collection instruments will be identified only by a unique study ID allocated to each baby. The place, date and time of the shift will also be anonymised by using only specific codes for observation shifts. Data on the paper-based observation checklists will be checked for completeness by a supervisor at the end of each day. The checklists will be double-entered into a custom-made database using Research Electronic Data Capture (REDCap) with inbuilt range and consistency checks. The entered data will be checked at the end of each day using pre-coded scripts for entry errors and completeness. Data will be exported for cleaning and analyses in Stata version 13 (Stata Corporation, Texas, USA).

Descriptive analysis will be undertaken on the pooled data across hospitals to determine the overall prevalence of care left undone and the common tasks that are left undone. We will also report the average number of tasks left undone per newborn and the common tasks left undone per newborn. Secondary analysis will be undertaken to explore variations in care done (or left undone) by the various sub-categories that will include: sector, nursing shift, nurse-patient ratios and category of the baby (disease severity).

Ethical considerations

The focus of our direct observations of care is what happens to newborns and we will not record any names or other identifying features of people who may be providing care to these newborns. We will only report pooled results stratified by sector, patient-nurse staffing ratios and category/severity of disease so as to preserve the confidentiality of the health facilities.

During the one-week familiarisation period, we will seek written individual informed consent from all nurses who will be providing care within the NBU. Nurses not available during this introductory period but who provide care during an observation shift will be asked for individual informed consent before the start of the observations. Additionally, at the beginning of every shift for which direct observations will be made, group verbal informed consent will be sought from nurses after an explanation of the study has been made (this is in addition to the 1-week familiarisation during which the study will be explained). We will also provide printed study briefs targeting health care providers explaining the study but also indicating that they are free to decline from being observed as an approach to on-going consent.

Written informed consent will be sought from the mothers of babies considered for direct observations at the beginning of each observation period. In cases where nurses or parents decline consent, babies under their care will not be considered for direct observation. It will be made clear that at any stage nurses or parents can withdraw consent/permission for observation, temporarily or for the rest of a shift, without explanation and with no penalty. Observations will only be undertaken where both the mother and nurses have provided consent to the study respectively.

For peer review only - http://bmjopen.bmj.com/site/about/guidelines.xhtml

Ethical approval for this study has been granted by the Kenya Medical Research Institute (KEMRI) Scientific and Ethics Review Unit (Approval No. KEMRI/SERU/CGMR-C/065/3404).

Dissemination of findings

An expert advisory group, including partners from the Ministry of Health, Nursing Council of Kenya, Nairobi City County, Kenya Medical Training College, Kenyatta University, and hospitals providing inpatient neonatal services has been involved in the development of the standards of nursing care being used to understand missed care in this study. This expert group has provided support for the study and is itself a key consumer of the results as these members are directly involved in policy making and training. This group will also provide a key channel for wider dissemination of the findings.

Highlighting the extent and magnitude of care left undone will provide important insights on the quality of care available to sick newborns, highlight human resource issues warranting attention and will likely influence recommendations on staffing norms and how care is organised and delivered in newborn units. Further, findings from this work will help guide the design of approaches and interventions for improving facility based care for this highly vulnerable population.

At the end of the study, findings will be provided to and discussed with participating hospitals and other relevant stakeholders. More widely, the international scientific community will be targeted via publications in peer reviewed journals as well as conferences.

Global public health relevance

Facility based care has been cited as one key approach to reduce neonatal mortality if evidence-based, high impact low cost interventions are appropriately delivered at high coverage[4]. Globally, improving quality of newborn care provided by facilities might save 600 000 small and ill neonates annually[4] while data from Kenya suggest that by 2030, 6000 newborn lives could be saved through the provision of childbirth and newborn care intervention packages alone[34].

The Kenyan government is promoting facility based delivery by making maternity care free for women. This has resulted in an increase in utilisation of maternal and newborn health services, potentially increasing the number of newborns accessing care in health facilities. At the same time significant challenges exist in nurse staffing and availability of wider resources[27] and this may limit any impact of increased access to care. The extent of nursing missed care in newborn units in LMIC and how it impacts on quality of care delivered has not previously been described. This is despite care in the newborn unit being heavily dependent on nurses. By characterising care left undone, we will identify important potential gaps in care delivered within newborn units that can inform discussions on how best to address these gaps and improve quality of care in Kenyan hospitals.

This study will be the first attempt, of which we are aware, to develop and apply observational missed care tools to understanding neonatal nursing care provision in LMIC. In fact a recent review by Jones and colleagues identified that only questionnaires have previously been used to quantify missed care[22]. An alternative method for data collection that we considered was the use of video in the newborn unit with later evaluation of what is done (or not done). However, informal discussions indicated this might be

For peer review only - http://bmjopen.bmj.com/site/about/guidelines.xhtml

controversial at this stage nonetheless this is a potential area for future research. We anticipate that this study will, therefore, be of global interest and provide an opportunity for application of the methods developed to other neonatal settings or in other disciplines to better understand nursing care provision and quality gaps. Further, findings of this work will be important in contributing to thinking on nurse staffing norms and how care is organised and delivered in LMIC newborn units influencing longer term policy on human resource planning. Most directly the findings will feed into the 'Kenya Task Sharing Policy and Guidelines for Health Care Services'[35], an initiative being led by Ministry of Health alongside other stakeholders as one of the ways of tackling health workforce shortages.

Authors' contributions

DG, ME and GAVM designed the study with contributions from RK and ET. DG, NA, and GS were responsible for the coordination and supervision of data collection. DG wrote the study protocol with substantial and critical input from all co-authors. All authors read and approved the final version of the manuscript.

Funding

This work was supported by the Health Systems Research Initiative joint grant provided by the Department for International Development, UK (DFID), Economic and Social Research Council (ESRC), Medical Research Council (MRC) and Wellcome Trust, grant number MR/M015386/1. ME is supported by a Wellcome Trust Senior Research Fellowship (#097170).

Competing interests

None

Figure legends

Figure 1: Steps for the sampling procedure

Multi-stage sampling procedure within a hospital for selecting newborns for direct observation

Figure 2: Precision levels for different newborn sub-populations and tasks

Estimated levels of precision for the different newborn sub-populations and tasks observed that the study will report since not all newborns observed will require all tasks .

For peer review only - http://bmjopen.bmj.com/site/about/guidelines.xhtml

1. References

1. WHO U. A Decade of Tracking Progress for Maternal, Newborn and Child Survival. 2015.

2. Lawn JE, Cousens S, Zupan J. 4 million neonatal deaths : When ? Where ? Why ? Lancet. 2005;365:9–18.

3. Belizán JM, McClure EM, Goudar SS, Pasha O, Esamai F, Patel A, et al. Neonatal death in low- to middle-income countries: a global network study. Am. J. Perinatol. 2012;29:649–56.

4. Bhutta ZA, Das JK, Bahl R, Lawn JE, Salam RA, Paul VK, et al. Can available interventions end preventable deaths in mothers, newborn babies, and stillbirths, and at what cost? Lancet (London, England). Elsevier; 2014;384:347–70.

5. Vesel L, Manu A, Lohela TJ, Gabrysch S, Okyere E, Ten Asbroek AHA, et al. Quality of newborn care: a health facility assessment in rural Ghana using survey, vignette and surveillance data. BMJ Open. 2013;3.

6. Mbwele B, Reddy E, Reyburn H. A rapid assessment of the quality of neonatal healthcare in Kilimanjaro region, northeast Tanzania. BMC Pediatr. 2012/11/23. 2012;12:182.

7. Opondo C, Ntoburi S, Wagai J, Wafula J, Wasunna A, Were F, et al. Are hospitals prepared to support newborn survival? - An evaluation of eight first-referral level hospitals in Kenya. Trop Med Int Heal. 2009/08/22. 2009;14:1165–72.

8. Dickson KE, Simen-Kapeu A, Kinney M V., Huicho L, Vesel L, Lackritz E, et al. Every Newborn: Health-systems bottlenecks and strategies to accelerate scale-up in countries. Lancet. 2014;384:438–54.

9. WHO. A universal Truth: No Health without a Workforce. 2014.

10. Wakaba M, Mbindyo P, Ochieng J, Kiriinya R, Todd J, Waudo A, et al. The public sector nursing workforce in Kenya: a county-level analysis. Hum. Resour. Health. 2014;12:6.

11. All-Party Parliamentary Group on Global Health. Triple Impact: How developing nursing will improve health, promote gender equality and support economic growth. 2016.

12. Reyburn H, Mwakasungula E, Chonya S, Mtei F, Bygbjerg I, Poulsen A, et al. Clinical assessment and treatment in paediatric wards in the north-east of the United Republic of Tanzania. Bull World Heal. Organ. 2008/02/26. 2008;86:132–9.

13. Gathara D, Opiyo N, Wagai J, Ntoburi S, Ayieko P, Opondo C, et al. Quality of hospital care for sick newborns and severely malnourished children in Kenya: A two-year descriptive study in 8 hospitals. BMC Health Serv. Res. 2011. p. 307.

14. Aluvaala J, Nyamai R, Were F, Wasunna A, Kosgei R, Karumbi J, et al. Assessment of neonatal care in clinical training facilities in Kenya. Arch. Dis. Child. 2014;archdischild-2014-306423-.

15. Schubert M, Glass TR, Clarke SP, Schaffert-Witvliet B, De Geest S. Validation of the Basel Extent of Rationing of Nursing Care instrument. Nurs. Res. 56:416–24.

16. Kalisch BJ. Missed nursing care: a qualitative study. J. Nurs. Care Qual. 21:306-13-5.

17. Lucero RJ, Lake ET, Aiken LH. Variations in nursing care quality across hospitals. J. Adv. Nurs. 2009;65:2299–310.

18. Ausserhofer D, Zander B, Busse R, Schubert M, De Geest S, Rafferty AM, et al. Prevalence, patterns

For peer review only - http://bmjopen.bmj.com/site/about/guidelines.xhtml

and predictors of nursing care left undone in European hospitals: results from the multicountry cross-sectional RN4CAST study. BMJ Qual. Saf. 2014;23:126–35.

19. Al-Kandari F, Thomas D. Factors contributing to nursing task incompletion as perceived by nurses working in Kuwait general hospitals. J. Clin. Nurs. 2009;18:3430–40.

20. Papastavrou E, Andreou P, Efstathiou G. Rationing of nursing care and nurse-patient outcomes: a systematic review of quantitative studies. Int. J. Health Plann. Manage. 2014;29:3–25.

21. Kalisch BJ, Tschannen D, Lee H. Missed Nursing Care, Staffing, and Patient Falls. J Nurs Care Qual. 2011;27:6–12.

22. Jones TL, Hamilton P, Murry N. Unfinished nursing care, missed care, and implicitly rationed care: State of the science review. Int. J. Nurs. Stud. Elsevier Ltd; 2015;52:1121–37.

23. Bekker M, Coetzee SK, Klopper HC, Ellis SM. Non-nursing tasks, nursing tasks left undone and job satisfaction among professional nurses in South African hospitals. J. Nurs. Manag. 2015;23:1115–25.

24. Lake ET, de Cordova PB, Barton S, Singh S, Agosto PD, Ely B, et al. Missed Nursing Care in Pediatrics. Hosp. Pediatr. American Academy of Pediatrics; 2017;7:378–84.

25. Tubbs-Cooley HL, Pickler RH, Younger JB, Mark B a. A descriptive study of nurse-reported missed care in neonatal intensive care units. J. Adv. Nurs. 2014;1–12.

26. Murphy GA V, Gathara D, Aluvaala J, Mwachiro J, Abuya N, Ouma P, et al. Nairobi Newborn Study: a protocol for an observational study to estimate the gaps in provision and quality of inpatient newborn care in Nairobi City County, Kenya. BMJ Open. 2016;6:e012448.

27. Murphy, Georgina A. V.; Gathara, David; Abuya, Nancy; Mwachiro, Jacintah; Ochola, Sam; Ayisi, Robert; English MHS that D for NEG. Can small and sick newborns requiring inpatient care access facilities equipped to offer essential services in a high mortality urban setting? - A cross-sectional study in Nairobi City County, Kenya. submitted.

28. Murphy, Georgina A. V.; Omondi Gregory B.; Gathara, David; Abuya, Nancy; Mwachiro, Jacintah; Kuria, Rose; Tallam Edna-Kimaiyo; English MNTAG. Expectations for nursing care in newborn units in Kenya – moving from implicit to explicit standards. BMJ Glob. Heal.

29. Gould JB, Qin C, Marks AR, Chavez G. Neonatal mortality in weekend vs weekday births. JAMA. 2003;289:2958–62.

30. Concha OP, Gallego B, Hillman K, Delaney GP, Coiera E. Do variations in hospital mortality patterns after weekend admission reflect reduced quality of care or different patient cohorts? A population-based study. BMJ Qual. Saf. 2014;23:215–22.

31. Kalisch BJ, Landstrom GL, Hinshaw AS. Missed nursing care: A concept analysis. J. Adv. Nurs. 2009;65:1509–17.

32. Leonard KL, Masatu MC. The use of direct clinician observation and vignettes for health services quality evaluation in developing countries. Soc. Sci. Med. 2005;61:1944–51.

33. Leonard K, Masatu MC. Outpatient process quality evaluation and the Hawthorne Effect. Soc. Sci. Med. 2006;63:2330–40.

34. Keats EC, Ngugi A, Macharia W, Akseer N, Khaemba EN, Bhatti Z, et al. Progress and priorities for

For peer review only - http://bmjopen.bmj.com/site/about/guidelines.xhtml

reproductive, maternal, newborn, and child health in Kenya: a Countdown to 2015 country case study. Lancet Glob. Heal. 2017;5:e782–95.

35. Ministry of Health. Task Sharing Policy Guidelines. 2017.

For peer review only

For peer review only - http://bmjopen.bmj.com/site/about/guidelines.xhtml

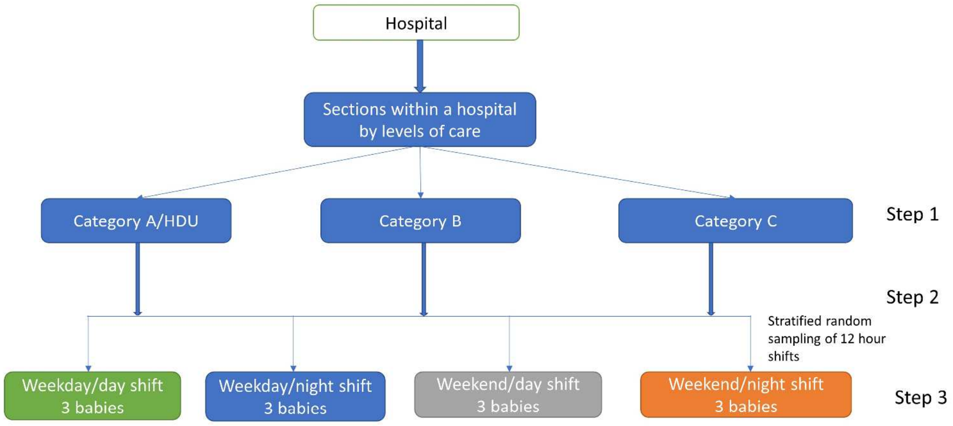

Multi-stage sampling procedure within a hospital for selecting newborns for direct observation

338x190mm (300 x 300 DPI)

review only

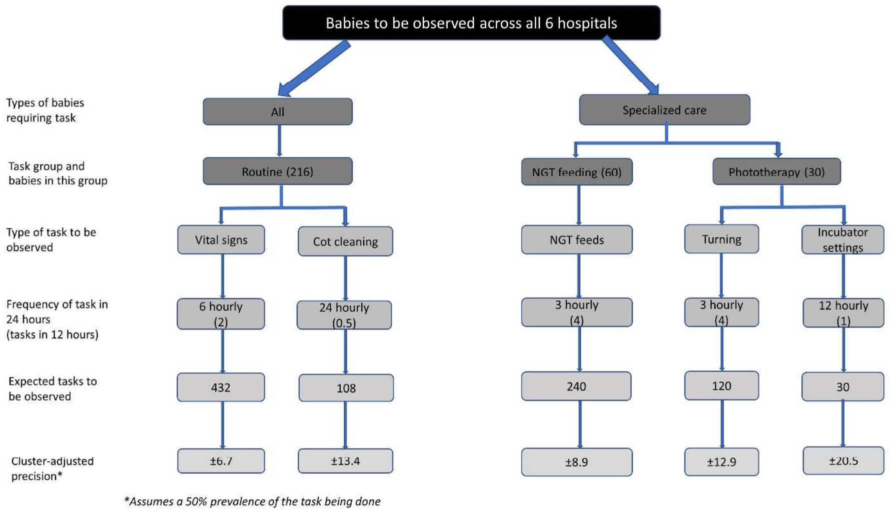

Precision levels for different newborn sub-populations and tasks

338x190mm (300 x 300 DPI)

BMJ Open

Quantifying nursing care delivered in Kenyan newborn units: Protocol for a cross-sectional direct-observational study

Journal:	*BMJ Open*
Manuscript ID	bmjopen-2018-022020.R1
Article Type:	Protocol
Date Submitted by the Author:	03-May-2018
Complete List of Authors:	Gathara, David; Kenya Medical Research Institute/Wellcome Trust Research Programme, Department of Public Health Research<br>Serem, George; Kenya Medical Research Institute/Wellcome Trust Research Programme, Department of Public Health Research<br>Murphy, Georgina ; Kenya Medical Research Institute/Wellcome Trust Research Programme, Department of Public Health Research; Bill and Melinda Gates Foundation<br>Abuya, Nancy; Kenya Medical Research Institute/Wellcome Trust Research Programme, Department of Public Health Research; Nairobi City County, Department of Curative and Preventative Services<br>Kuria, Rose; Kenya Medical Training College, Department of Nursing<br>Tallam, Edna; Nursing Council of Kenya, Department of Registration and Licensing<br>English, Mike; University of Oxford, Nuffield Department of Medicine and Department of Paediatrics; Kenya Medical Research Institute/Wellcome Trust Research Programme, Department of Public Health Research
**Primary Subject Heading**:	Nursing
Secondary Subject Heading:	Health services research, Research methods
Keywords:	Health & safety < HEALTH SERVICES ADMINISTRATION & MANAGEMENT, NEONATOLOGY, STATISTICS & RESEARCH METHODS

SCHOLARONE™
Manuscripts

Quantifying nursing care delivered in Kenyan newborn units: Protocol for a cross-sectional direct-observational study

Investigators and Institutional Affiliations

David Gathara[1*], George Serem[1], Georgina A. V. Murphy [1, 2], Nancy Abuya[1,3], Rose Kuria[4], Edna Tallam[5], Mike English [1,2]

Affiliations

1. Department of Public Health Research, Kenya Medical Research Institute/Wellcome Trust Research Programme, Nairobi, Kenya
2. Nuffield Department of Medicine and Department of Paediatrics, University of Oxford, UK
3. Department of Curative and Preventative services, Nairobi City County, Kenya
4. Department of Nursing, Kenya Medical Training College, Kenya
5. Department of Registration and Licensing, Nursing Council of Kenya

*Corresponding author
Dr David Gathara
P.O Box 43640 00100
Kenya Medical Research Institute/Wellcome Trust Research Programme
Nairobi, Kenya
Email: Dgathara@kemri-wellcome.org
Phone: (+254) 721659321; (+254) 730 162 000

Abstract

Introduction: In many African countries, including Kenya, a major barrier to achieving child survival goals is the slow decline in neonatal mortality that now represents 45% of the under-5-mortality. In newborn care, nurses are the primary care givers in newborn settings and are essential in the delivery of safe and effective care. However, due to high patient workloads and limited resources, nurses may often consciously or unconsciously prioritise tasks and the care they provide resulting in some tasks being left undone or partially done (missed care). Missed care has been associated with poor patient outcomes in high income countries. However, missed care, examined by direct observation, has not previously been the subject of research.

Methods and analysis: The aim of this study is to quantify essential neonatal nursing care provided to newborns within newborn units. We will undertake a cross-sectional study utilising direct-observational methods within newborn units in 6 health facilities in Nairobi City County across the public, private-for-profit and private-not-for-profit sectors. A total of 216 newborns will be observed between 1st September 2017 to 30th May 2018. Stratified random sampling will be used to select random 12-hour observation periods while purposive sampling will be used to identify newborns for direct-observation.

For peer review only - http://bmjopen.bmj.com/site/about/guidelines.xhtml

We will report the overall prevalence of care left undone, the common tasks that are left undone and describe any sharing of tasks with people not formally qualified to provide care.

Ethics and dissemination: Ethical approval for this study has been granted by the Kenya Medical Research Institute Scientific and Ethics Review Unit. Written informed consent will be sought from mothers and nurses. Findings from this work will be shared with the participating hospitals, an expert advisory group that comprises of members involved in policy making and more widely to the international community through conferences and peer-reviewed journals.

Strengths and limitations

- The use of direct observational methods to quantifying nursing care delivered or left undone an approach that has not been previously used in LMICs.
- Different sectors (public, private-for-profit, private-not-for-profit) in health service provision have been included in this study.
- The study provides a 24-hour assessment of neonatal nursing care provision including care provided on weekends and weekdays.
- Our scope is limited by the few number of newborns to be observed within each hospital.
- Despite our efforts to minimize the Hawthrone effect, we cannot rule out the possibility of nurses changing the way they provided care during the observational periods.

Introduction

Despite progress globally, most African countries including Kenya have made insufficient progress in reducing child mortality. In most countries, this can be partly attributed to only slow declines in neonatal mortality. As a consequence, about 45% of mortality for children under-5 years is attributable to neonatal mortality[1]. Of these neonatal deaths, approximately 75% occur in the first 7 days of life and half of these within 24 hours of life[2][3]. A recent review by Bhutta and colleagues indicated that high impact low cost interventions could avert more than 71% of neonatal deaths with 82% of this effect being attributable to facility-based care[4]. However, reports from low income settings highlight that the quality of newborn care in health facilities is often poor[5][6][7]. Therefore, strengthening the quality of facility based care for newborns will be essential in improving newborn outcomes.

Human Resources for Health (HRH) inadequacies is a major factor limiting delivery of quality neonatal services [8]. Globally, the shortage of health workers is estimated currently at over 7 million and by 2035 the deficit is estimated to be 12.9 million[9]. The shortages in the available workforce are worst in developing countries where inequitable distribution of available health workers may compound the problems. In Kenya, Wakaba and colleagues reported that public sector nursing densities ranged between 0.008 to 1.2 per 1000 population across counties[10] compared to an internationally suggested

For peer review only - http://bmjopen.bmj.com/site/about/guidelines.xhtml

minimum health workforce threshold of 2.5/1000 population for doctors, nurses and midwives. In Nairobi County, the nurse densities ranged between 0.21 to 0.40 per 1000 population[10].

There is little specific exploration of the impact of nursing workforce shortfalls on inpatient care in Low and Middle-Income Countries (LMIC). Yet to improve quality of care it is essential that we understand who delivers care (what tasks are done by whom), how care is delivered (how are tasks performed) and critically analyse what tasks are left undone. In most health systems, nurses are gatekeepers of the health care being delivered. They are vested with the responsibility of delivering interventions prescribed by other providers (doctors, nutritionists etc) in addition to providing nurse initiated interventions[11]. As a consequence, few interventions reach the patient without the involvement of the nurse. Yet the few existing evaluations of the quality of care provided to newborns in LMIC have focused on the more medical aspects of care[6,12,13][14]. In LMIC facilities, large patient workloads, insufficient staff and resources, urgent patient situations, and unexpected rises in patient volume and/or acuity on the unit (among other factors) might result in all facets of nursing care being delayed or neglected. This phenomenon has been described as 'implicit rationing'[15], 'missed care'[16], or 'unmet nursing care needs'[17], 'care left undone'[18], or 'task incompletion'[19]. Hereafter, we use the term missed care to encompass all of these terms. Such missed care may have a particularly devastating impact on outcomes in newborn units where nurses are the primary care givers to this highly dependent group.

Justifying a focus on missed nursing care several studies have reported associations between missed care and patient outcomes[20,21][22]. Although there is a growing literature on missed care the majority is from high resource settings, with only one study in South Africa[23] providing an early formal attempt to quantify the extent of the problem in this middle income country. Furthermore, almost all the literature on missed care is based on nurse surveys with only two focusing on newborn care provision[24,25]. Although, nurse surveys on missed care have proven useful, there is a call to undertake more research with a special focus on objective observational methods as no studies of this type were identified in a recent systematic review [22]. The proposed study aims to characterise the care delivered (tasks done or left undone and who does these tasks) to newborns receiving care within newborn units in Nairobi, Kenya by making direct observations of care being provided. This will provide in-depth and objective insights on missed care with particular reference to locally agreed standards for nurses providing neonatal care.

Methods and analysis

This is a cross-sectional study that will involve direct observation of care provided to individual newborns with the aim of describing the essential neonatal nursing care given or missed within newborn units. It will be undertaken in 6 hospitals in Nairobi City County, Kenya, in the period 1st September 2017 to 30th May 2018.

Study site

The proposed research work will be undertaken as part of a broader set of work being conducted in collaboration with Nairobi City County. This collaboration includes work to characterise all the facilities providing inpatient newborn care 24 hours, 7 days a week (hereafter referred to as 24/7) in Nairobi [26],

For peer review only - http://bmjopen.bmj.com/site/about/guidelines.xhtml

quality of clinical care provided newborns[27], and ethnographic work to understand the wider context and practice of neonatal nursing. Based on findings from the broader study, Nairobi county has 34 health facilities providing 24/7 inpatient newborn care, of these 2 health facilities declined to take part in prior quality of care surveys[27]. Excluding the military health facility with restrictive admission policy, the remaining 31 health facilities that form the population for this study provide 99% of all inpatient neonatal care.

This current study will focus on primary referral (secondary care) facilities that have more than 100 neonatal admissions annually. Thirteen facilities meet these criteria and together provide care to over 96% of the sick-newborn population accessing care within Nairobi County[27]. These 13 facilities will be stratified by workload (newborn admissions per year <=500 low; >500 high) and six health facilities purposefully selected to ensure representation of 2 hospitals in each of the public, private-not-for profit and private-for-profit sectors, with one high and one low workload facility in each sector. Purposeful selection will be used as it is important in this sensitive and innovative work to have the strong support of the hospital administration. This initial work will therefore help illustrate the nature and magnitude of the challenge of missed care but not make claims to provide a statistically representative picture of missed care which would be challenging given the great diversity of facilities found in Nairobi, with some health facilities having as low as 10 neonatal admissions per year[27].

Study populations

All newborns admitted within the newborn unit in the 6 selected health facilities over the period of the study in each facility will form the potential study population. However, newborns meeting the following exclusion criteria will not be observed: i) newborns requiring specialised treatment to whom the draft minimum nursing standards for neonatal care[28] may not be applicable, for instance newborns with gross malformations or those receiving post-operative care, ii) newborns who are critically ill and at risk of death within a 12-hour observation period as defined by the clinician in charge of the newborn unit for whom observation might cause distress to families, iii) newborns for whom guardians do not provide consent, and iv) newborns receiving care from nurses or guardians who decline to be observed in the care provision process.

Sampling procedures

To describe the care being provided to newborns admitted in newborn units and the spectrum of inpatient services they receive, we are aiming to sample time in 12-hour shifts randomly using the steps described in figure 1.

Figure 1: Steps for the sampling procedure

Step 1

Care within the newborn unit can be organised in a way that babies requiring different levels of care are in different sections/rooms. Intensive care employing invasive mechanical ventilation is not available outside tertiary hospitals in Kenya and thus definitions for different levels of newborn care provided by County Hospitals were adopted. These are taken from draft nursing standards for neonatal care that

For peer review only - http://bmjopen.bmj.com/site/about/guidelines.xhtml

categorise newborn unit sections (and thus babies) in the following way[28]. **Category A (high dependency unit, HDU)**: Babies on oxygen/CPAP and intravenous fluids who are often acutely ill and unstable and require the closest monitoring. **Category B**: Babies who have stabilized but may still be ill and receiving, for example, assisted feeding (nasal-gastric feeds), intravenous drugs; or being observed for convulsions or apnoea. **Category C**: Babies who are quite stable who should be receiving KMC, or stable abandoned babies, or recovering babies requiring completion of treatment such as the last doses of antibiotics or transitioning to oral feeding. Our primary sampling strategy will be based on identifying these organisational sub-sections (Category A; B; C rooms or incubator/cot spaces) in each facility. Where facilities have no clear organisational demarcation into Category A, B and C sub-sections, we will adapt the observations to suit the organisation of care in each facility. However, we will endeavour to identify and select, for observation, newborns with varying degrees of illness severity who would be classified as meeting criteria for Category A, B or C in such settings.

Step 2

In each hospital, stratified-random sampling will be used to generate a random sample of twelve shifts/time blocks of 12 hours (144 observation hours per hospital) from within a 3 week period stratified by disease severity (category A, B, C), weekdays and weekends as well as night and day shifts as care has been shown to vary across these periods[29,30]. The 12 shifts will be divided equally across the newborn unit sections within a hospital where there is more than one section. Where all babies are cared for in one room without clear sub-sections all observation periods will be conducted in this same setting with efforts to observe babies requiring different levels of care.

Step 3

We feel that it is logistically feasible for one observer to make direct observations of 3 babies located in adjacent cots in the same ward area at one time. Therefore, for each shift and section, 3 babies who meet the inclusion criteria will be purposefully selected to ensure babies are in one ward area and within close proximity to allow direct observations. In smaller units where the number of babies per section may be less than 3, we will observe all the babies available in the section and classify them as eligible for care in category A, B or C.

Sample size

This is an exploratory cross-sectional study and as such, we illustrate the precision with which we can report proportions of tasks done (or not done) assuming different sized denominators. The size of the denominator is related to the recommended frequency that tasks should be performed (see figure 2). To estimate the precision of reporting we have used a sample estimation approach for cluster designs and assuming a design effect of 2 to adjust for clustering of observed tasks around individual newborns within hospitals.

At most we expect to recruit 216 newborns across 6 hospitals (36 newborns per hospital with 3 babies for each of the 12 shifts/time blocks). For tasks that would be conducted with an expected frequency of once in 12 hours (e.g. intravenous pencillin administration) we might therefore observe 216 task

For peer review only - http://bmjopen.bmj.com/site/about/guidelines.xhtml

opportunities. Taking a (statistically) conservative assumption that on 50% of expected occasions the task is observed to be done then we could report this with a precision of ± 13.4%. Similarly, if a task should be conducted every 6 hours then the denominator would be 432 expected tasks and if half were observed the precision of this estimate would be ± 6.7%.

Not all babies observed will require all tasks. For example, some babies may not be receiving intravenous drugs. This will reduce the effective size of the denominator and reduce the precision we can report. Similarly, if babies have to leave the area of observation (for example they are moved to a new ward area or are sent for X-ray etc) this may reduce the number of expected tasks that can be observed. In addition, failure to recruit 3 babies at each 12-hour shift at each facility may also reduce the number of tasks observed. We illustrate the effect this has on the precision of reported estimates in figure 2, which illustrates that with as few as 30 expected tasks, reporting a precision of ± 20.5% is possible.

As this work is a first of its kind (globally as far as we are aware) we feel that providing estimates of the proportion of tasks done/left undone with a precision of approximately ± 20% will be sufficient to provide valuable insights into the challenges faced by nurses in providing newborn care.

Figure 2: Precision levels for different newborn sub-populations and tasks

Procedures

For each newborn being observed, the diagnosis and disease severity information will be collected from medical records as this informs the expected number of tasks. At the beginning of every shift for which observations will be made, the total number of nursing tasks expected to be delivered will be determined with reference to the medical and nursing records (for disease severity and specific interventions like phototherapy) and general aspects of care each baby should receive. For each of the newborns selected to participate in the study, we will make direct observations on how often certain routine nursing tasks (listed in table 1) are undertaken in a 12-hour shift (7am -7pm or 7pm to 7am) using an observation checklist. Observations will be stopped if a baby is transferred out of a section or discharged, however, we will use the data collected up to the point of exit and the number of observation hours will be documented. If the babies' condition changes and their category changes but they remain in the same observation area, we will document this change and revise the expected number of tasks. In both instances the effective denominator for expected nursing tasks will be changed as required.

The observer will be stationed in a ward area where they do not obstruct care provision but can observe the care being provided to the newborns selected for observation. Because most of the documentation activities happen at the nurses' desk/station, all tasks related to documentation cannot be observed from the cot-side. The documented tasks for the babies under observation will be evaluated at the end of each 12-hour shift for which observations will be made. We acknowledge that 12 hours is a long period and the observer's efficiency for making observations might reduce as time progresses within an observation time block and study period. We will factor in rest periods in the 12-hour shifts that coincide with when nurses take their breaks, for instance, during tea and lunch breaks, periods which we

For peer review only - http://bmjopen.bmj.com/site/about/guidelines.xhtml

anticipated limited or no tasks will be undertaken. Further, to allow enough rest between observation time blocks, we will aim to have a maximum of 3, 12-hour observation periods per week per observer with at least a 24-hour rest period between observations. The 12-hour periods were selected because they span nursing shift change overs that allowed documentation of care round-the-clock and made random selection of time blocks more feasible.

The observation checklist (supplementary file 1) is based on nursing standards produced after two expert group meetings held in November 2015 and July 2016 at the KEMRI Wellcome Trust Research Programme offices. The nurse stakeholder group comprised expert nurses who are in the teaching profession or senior practitioners and including the acting Chief Nursing Officer for Kenya. They defined a minimum standard for performing nursing tasks on newborn units[28]. A sub-set of tasks identified as critical (listed in table 1) by the nurse stakeholder group was explicitly marked as to only be performed by nurses and not by other personnel due to the skills required when they are delivered. Where necessary tasks are broken down into manageable observable task components to facilitate observation. For instance, naso-gastric feeding is broken down into insertion of the NGT (as required), checking for gastric aspirate before feeding, counter checking feed volumes to be given with prescription, actual feeding and charting the feeds given. The purpose of observation is not to assess how well any particular aspect of a task is done (eg. the care taken in administering naso-gastric feeds) but simply to determine if the task (or task component) was (or was not) done at all.

Prior to the start of the study, the observation checklist (supplementary file 1) will be extensively piloted over a period of 6 weeks to determine the quantity and quality of data that can be reasonably gathered and will be adapted as needed. The tool will be piloted in one public health facility that will not be used as a study site in the final study by the research assistant who will subsequently be responsible for training the data clerks. During piloting, we will observe care provision in each of the nursing 12-hour time blocks to explore what tasks can be observed, what number of newborns will be logistically feasible to observe, the different nursing routines in the different shifts and the documents used for reporting on nursing activities.

Table 1: Routine and critical tasks for observation

Routine tasks	Critical tasks
Patient assessment at the beginning of each shift<br>Cleaning of the baby<br>Changing baby's linen<br>Changing the baby's position<br>Checking incubator settings<br>Ward round attendance and active note taking<br>Weighing<br>Elimination care<br>Communication/counselling parents<br>Cord care<br>Vital signs measurement	Naso-gastric feeding<br>• Insertion of the NGT<br>• Testing whether it is in the correct position<br>• Checking for gastric aspirate before feeding<br>• Preparation of feeds and counter checking feed volumes<br>• Actual feeding and charting the feeds<br>Intravenous drug/fluid administration<br>• Reviewing treatment sheet<br>• Checking cannula sites – care<br>• Regulating flow<br>• Input/output charting for fluids<br>• Document treatments given

For peer review only - http://bmjopen.bmj.com/site/about/guidelines.xhtml

• Pulse rate<br>• Temperature<br>• Respiratory rate<br>• Oxygen saturation<br>Documentation<br>• Updating the nursing cardex<br>• Discharge and admission registration	Oxygen therapy<br>• Fixing of oxygen/nasal prongs<br>• Checking tube position and nostril –care, damage<br>• Initiating and regulating oxygen flow<br>• Documenting oxygen treatment<br>Photo therapy<br>• Baby positioning<br>• Placing/checking eye pad is in place<br>• Checking eyes for damage<br>• Checking and monitoring phototherapy settings<br>• Documenting of phototherapy<br>Support for KMC

For purposes of this study, we adopted the definition of missed care reported in wider literature[22,31], and was therefore defined as care that the nursing advisory group regard as necessary (primarily essential neonatal nursing tasks) as part of routine newborn care that are left undone or are delivered by any person other than the nurse or a qualified health care provider (nutritionist, doctor, clinical officer etc). However, tasks that will be done by a senior student nurse under direct observation/supervision by the nurse will be considered as done and documented as not done if no supervision is observed. For example, tasks done by a senior student nurse who is being supervised to conduct naso-gastric feeding by a qualified nurse in the room at the time and focused on the supervision will be regarded as done. If the same student does the task while the nurse supervising is in a different part of the ward it will be regarded as not done (with a record of who completed the task made). Additional data to be collected related to each block of observation time will include data on the nurse-patient ratio for the ward as a whole, what additional staff are present within the unit e.g. nursing students, support staff etc. and patient workloads within the different sections of the ward.

Practical considerations for undertaking direct observations

Observations will be made by a person familiar with the hospital environment, equipment, processes (like ward rounds) and language but who is not a nurse or clinician (doctor/clinical officer). Given the potential sensitivity of this form of observation, it is important that this person is considered a professional rather than an 'outsider' (who might not be bound by professional codes of confidentiality). Having an observer who has an understanding of the setting but who is not a clinician or nurse may also overcome problems of the observer making judgements about what is being observed based on their own standards of practice or being influenced as they make observations by professional allegiances (eg. a nurse observer may not wish to record that a task is left undone). There might also be ethical challenges for an observer with a clinical/nurse background who might feel obliged to intervene in the provision of care or become co-opted to complete tasks. An observer who is not licensed to offer the form of interventions/care being given to babies will still however be able to report to health care providers within the unit in the event they identify gaps or situations in which

For peer review only

For peer review only - http://bmjopen.bmj.com/site/about/guidelines.xhtml

newborns are put at risk. For instance, if they see a newborn convulsing or vomiting, they will alert the nurse. Suitable backgrounds for observers might include nutritionists, health records officers, ward based clerical workers, or laboratory or pharmacy staff with at least two-year's experience within hospital settings. In this study nutritionists will be recruited to undertake the observations.

We will recruit six observers who meet the above criterion. The observers will be trained for a period of 2 weeks by one of the study personnel who has a nursing background and has 2 years' experience providing care to newborns within a newborn unit. Standard operating procedures (SOPs) will be developed and will serve as a guide for the observational work. During the training period, the observers and the trainer will do observations and comparisons will be made. Any differences will be discussed and where necessary the SOPs will be revised to improve on clarity. Supervision during the data collection period will be undertaken by study staff with a nursing background with routine weekly reviews of the observation checklists for completion and consistency.

To reduce the Hawthorne effect where nurses might change the way they provide care when they are being observed, the observer will spend at least 1 week in the health facility before starting the observational work. This will also allow him/her to familiarise themselves with the environment, to explain the study to staff and parents and for the staff within the newborn unit to get used to them. In addition, the observer will make it clear that the observations are not an assessment but a means of understanding what care is possible to provide given existing resource constraints. Formal study observations will then begin in the period after the one-week familiarisation period. This approach is supported by evidence that health care providers change their practice slightly when the observations start but these changes are short-lived and quickly dissipate with health care providers soon reverting to their previous practice[32,33]. In addition, the 12-hour observation periods are randomly generated and the hospitals will not be aware of the day, shift or category of babies the observer would next come to do observations for.

Practical and public involvement

Patients and the public were not involved in the development of the research question or outcome measures. However, as part of the broader set of work within which this study is embedded, there is ongoing work to understand patient experiences focusing on experiences of mothers with newborns admitted within the newborn unit. We hope findings from our study will compliment those from the patient experiences work and will provide insights on aspects of care that are important to mothers but also inform the design of interventions to improve care.

Data Management and analysis

Data will be collected on paper-based observation checklists, one for each baby that is the focus of observation. No participant identifiable data will be collected and data collection instruments will be identified only by a unique study ID allocated to each baby. The place, date and time of the shift will also be anonymised by using only specific codes for observation shifts at data entry. Data on the paper-based observation checklists will be checked for completeness by a supervisor at the end of each day. The checklists will be double-entered into a custom-made database using Research Electronic Data Capture

For peer review only - http://bmjopen.bmj.com/site/about/guidelines.xhtml

(REDCap) with inbuilt range and consistency checks. The entered data will be checked at the end of each day using pre-coded scripts for entry errors and completeness. Data will be exported for cleaning and analyses in Stata version 13 (Stata Corporation, Texas, USA).

Descriptive analysis will be undertaken on the pooled data across hospitals to determine the overall prevalence of care left undone and the common tasks that are left undone. We will also report the average number of tasks left undone per newborn and the common tasks left undone per newborn. Secondary analysis will be undertaken to explore variations in care done (or left undone) by the various sub-categories that will include: sector, nursing shift, nurse-patient ratios and category of the baby (disease severity).

Ethical considerations

The focus of our direct observations of care is what happens to newborns and we will not record any names or other identifying features of people who may be providing care to these newborns. We will only report pooled results stratified by sector, nurse-patient staffing ratios and category/severity of disease so as to preserve the confidentiality of the health facilities.

During the one-week familiarisation period, we will seek written individual informed consent from all nurses who will be providing care within the newborn unit. Additional consent forms and study briefs will be left in the ward to allow nurses not available during this introductory period but who provide care in the newborn unit to review and indicate their willingness to participate. During an observation shift these nurses will be asked for individual informed consent before the start of the observations. Additionally, at the beginning of every shift for which direct observations will be made, group verbal informed consent will be sought from nurses after an explanation of the study has been made (this is in addition to the 1-week familiarisation during which the study will be explained). We will also provide printed study briefs targeting health care providers explaining the study but also indicating that they are free to decline from being observed as an approach to on-going consent.

Written informed consent will be sought from the mothers of babies considered for direct observations at the start of each observation period. In Kenya, pregnant adolescents between ages (15 – 17 years) are considered 'emancipated minors' and their written informed consent will be obtained[34–36]. The start of the direct observation shift (7am or 7pm) is just before when mothers are in the newborn unit for the 3-hourly feeding session at 6am or 6pm, as such mothers will be approached during this period. In instances, where mothers indicate more time is required to make a decision or to consult they will be allowed to do so and another mother whose baby meets the criteria for observation will be approached. In cases where nurses or parents decline consent, babies under their care will not be considered for direct observation. It will be made clear that at any stage nurses or parents can withdraw consent/permission for observation, temporarily or for the rest of a shift, without explanation and with no penalty. Observations will only be undertaken where both the mother and nurses have provided consent to the study respectively.

Ethical approval to conduct this study in all hospitals has been granted by the Kenya Medical Research Institute (KEMRI) Scientific and Ethics Review Unit (Approval No. KEMRI/SERU/CGMR-C/065/3404) while permission to undertake the study in the respective hospitals was sought from each of the hospital's administrative offices.

For peer review only - http://bmjopen.bmj.com/site/about/guidelines.xhtml

Dissemination of findings

An expert advisory group, including partners from the Ministry of Health, Nursing Council of Kenya, Nairobi City County, Kenya Medical Training College, Kenyatta University, and hospitals providing inpatient neonatal services has been involved in the development of the standards of nursing care being used to understand missed care in this study. This expert group has provided support for the study and is in itself a key consumer of the results as these members are directly involved in policy making and training. This group will also provide a key channel for wider dissemination of the findings.

Highlighting the extent and magnitude of care left undone will provide important insights on the nursing care available to newborns admitted within newborn units, highlight human resource issues warranting attention and will likely influence recommendations on staffing norms and how care is organised and delivered in newborn units. Further, findings from this work will help guide the design of approaches and interventions for improving facility based care for this highly vulnerable population.

At the end of the study, findings will be provided to and discussed with participating hospitals and other relevant stakeholders. More widely, the international scientific community will be targeted via publications in peer reviewed journals as well as conferences.

Global public health relevance

Facility based care has been cited as one key approach to reduce neonatal mortality if evidence-based, high impact low cost interventions are appropriately delivered at high coverage[4]. Globally, improving quality of newborn care provided by facilities might save 600 000 small and ill neonates annually[4] while data from Kenya suggest that by 2030, 6000 newborn lives could be saved through the provision of childbirth and newborn care intervention packages alone[37].

The Kenyan government is promoting facility based delivery by making maternity care free for women. This has resulted in an increase in utilisation of maternal and newborn health services, potentially increasing the number of newborns accessing care in health facilities. At the same time significant challenges exist in nurse staffing and availability of wider resources[27] and this may limit any impact of increased access to care. The extent of missed nursing care in newborn units in LMIC and how it impacts on quality of care delivered has not previously been described. This is despite care in the newborn unit being heavily dependent on nurses. By characterising care left undone, we will identify important potential gaps in care delivered within newborn units that can inform discussions on how best to address these gaps and improve quality of care in Kenyan hospitals.

This study will be the first attempt, of which we are aware, to develop and apply direct observational missed care tools to understanding neonatal nursing care provision in LMIC. In fact, a recent review by Jones and colleagues identified that only questionnaires have previously been used to quantify missed care and these might be limited by reporting bias[22]. However, we acknowledge that direct observational methods have limitations on the number and actual tasks that can be observed, might be influenced by observer bias and are at risk of Hawthorne effect. As such, the tasks in our observation checklist (supplementary file 1)_are limited to essential neonatal nursing tasks provided at the bedside that can be observed while those linked to documentation will involve review of medical records at the

For peer review only - http://bmjopen.bmj.com/site/about/guidelines.xhtml

nurses' desk since documentation of care is done at the nurses' desk and not at the cot-side. Moreover, observing the documentation of a task or reviewing records for evidence of documentation is likely to provide similar results. An alternative method for data collection that we considered was the use of video in the newborn unit with later evaluation of what is done (or not done). However, informal discussions indicated this might be controversial at this stage due to the ethical and medico-legal issues that might emanate from this approach and the administrative approvals required for video recording in hospitals, nonetheless this is a potential area for future research. The selection of health facilities was purposive, as support by the hospital administration was important due to the nature of data collection and the sensitivity associated with direct observation of care. As such, we cannot rule out selection bias as facilities providing better care might have been more likely to agree to partake in the study. However, this is the first such study using direct observational methods to quantify missed care and in a LMIC and therefore the findings will still be important. We anticipate that this study will, therefore, be of global interest and provide an opportunity for application of the methods developed to other neonatal settings or in other disciplines to better understand nursing care provision and quality gaps. Further, findings of this work will be important in contributing to thinking on nurse staffing norms and how care is organised and delivered in LMIC newborn units. This will be crucial in influencing longer term policy on human resource planning. Most directly the findings will feed into the 'Kenya Task Sharing Policy and Guidelines for Health Care Services'[38], an initiative being led by Ministry of Health alongside other stakeholders as one of the ways of tackling health workforce shortages.

Authors' contributions

DG, ME and GAVM designed the study with contributions from RK and ET. DG, NA, and GS were responsible for the coordination and supervision of data collection. DG wrote the study protocol with substantial and critical input from all co-authors. All authors read and approved the final version of the manuscript.

Funding

This work was supported by the Health Systems Research Initiative joint grant provided by the Department for International Development, UK (DFID), Economic and Social Research Council (ESRC), Medical Research Council (MRC) and Wellcome Trust, grant number MR/M015386/1. ME is supported by a Wellcome Trust Senior Research Fellowship (#097170).

Competing interests

None

Figure legends

Figure 1: Steps for the sampling procedure

Multi-stage sampling procedure within a hospital for selecting newborns for direct observation

For peer review only - http://bmjopen.bmj.com/site/about/guidelines.xhtml

420 Figure 2: Precision levels for different newborn sub-populations and tasks

421 Estimated levels of precision for the different newborn sub-populations and tasks observed that the
422 study will report since not all newborns observed will require all tasks.

For peer review only

For peer review only - http://bmjopen.bmj.com/site/about/guidelines.xhtml

1. References

1. World Health Organization. Countdown to 2015: A Decade of Tracking Progress for Maternal, Newborn and Child Survival. Geneva; 2015.

2. Lawn JE, Cousens S, Zupan J. 4 million neonatal deaths : When ? Where ? Why ? Lancet. 2005;365:9–18.

3. Belizán JM, McClure EM, Goudar SS, Pasha O, Esamai F, Patel A, et al. Neonatal death in low- to middle-income countries: a global network study. American Journal of Perinatology. 2012;29:649–56.

4. Bhutta ZA, Das JK, Bahl R, Lawn JE, Salam RA, Paul VK, et al. Can available interventions end preventable deaths in mothers, newborn babies, and stillbirths, and at what cost? Lancet (London, England). Elsevier; 2014;384:347–70.

5. Vesel L, Manu A, Lohela TJ, Gabrysch S, Okyere E, Ten Asbroek AHA, et al. Quality of newborn care: a health facility assessment in rural Ghana using survey, vignette and surveillance data. BMJ Open. 2013;3.

6. Mbwele B, Reddy E, Reyburn H. A rapid assessment of the quality of neonatal healthcare in Kilimanjaro region, northeast Tanzania. BMC Pediatrics. 2012/11/23. 2012;12:182.

7. Opondo C, Ntoburi S, Wagai J, Wafula J, Wasunna A, Were F, et al. Are hospitals prepared to support newborn survival? - An evaluation of eight first-referral level hospitals in Kenya. Tropical Medicine and International Health. 2009/08/22. 2009;14:1165–72.

8. Dickson KE, Simen-Kapeu A, Kinney M V., Huicho L, Vesel L, Lackritz E, et al. Every Newborn: Health-systems bottlenecks and strategies to accelerate scale-up in countries. Lancet. 2014;384:438–54.

9. Campbell J, Dussault G, Buchan J, Pozo-Martin F, Arias MG, Leone C, et al. A universal Truth: No Health without a Workforce. World Health Organization, Geneva; 2013.

10. Wakaba M, Mbindyo P, Ochieng J, Kiriinya R, Todd J, Waudo A, et al. The public sector nursing workforce in Kenya: a county-level analysis. Human Resources for Health. 2014;12:6.

11. All-Party Parliamentary Group on Global Health. Triple Impact: How developing nursing will improve health, promote gender equality and support economic growth. 2016.

12. Reyburn H, Mwakasungula E, Chonya S, Mtei F, Bygbjerg I, Poulsen A, et al. Clinical assessment and treatment in paediatric wards in the north-east of the United Republic of Tanzania. Bull World Health. Organ. 2008/02/26. 2008;86:132–9.

13. Gathara D, Opiyo N, Wagai J, Ntoburi S, Ayieko P, Opondo C, et al. Quality of hospital care for sick newborns and severely malnourished children in Kenya: A two-year descriptive study in 8 hospitals. BMC Health Services Research. 2011. p. 307.

14. Aluvaala J, Nyamai R, Were F, Wasunna A, Kosgei R, Karumbi J, et al. Assessment of neonatal care in clinical training facilities in Kenya. Archives of Disease in Childhood. 2014;archdischild-2014-306423-.

15. Schubert M, Glass TR, Clarke SP, Schaffert-Witvliet B, De Geest S. Validation of the Basel Extent of Rationing of Nursing Care instrument. Nursing Research. 56:416–24.

16. Kalisch BJ. Missed nursing care: a qualitative study. Journal of Nursing Care Quality. 21:306-13-5.

17. Lucero RJ, Lake ET, Aiken LH. Variations in nursing care quality across hospitals. Journal of Advanced Nursing. 2009;65:2299–310.

For peer review only - http://bmjopen.bmj.com/site/about/guidelines.xhtml

For peer review only

18. Ausserhofer D, Zander B, Busse R, Schubert M, De Geest S, Rafferty AM, et al. Prevalence, patterns and predictors of nursing care left undone in European hospitals: results from the multicountry cross-sectional RN4CAST study. BMJ Quality and Safety. 2014;23:126–35.

19. Al-Kandari F, Thomas D. Factors contributing to nursing task incompletion as perceived by nurses working in Kuwait general hospitals. Journal of Clinical Nursing. 2009;18:3430–40.

20. Papastavrou E, Andreou P, Efstathiou G. Rationing of nursing care and nurse-patient outcomes: a systematic review of quantitative studies. International Journal of Health Planning and Management. 2014;29:3–25.

21. Kalisch BJ, Tschannen D, Lee H. Missed Nursing Care, Staffing, and Patient Falls. Journal of Nursing Care Quality. 2011;27:6–12.

22. Jones TL, Hamilton P, Murry N. Unfinished nursing care, missed care, and implicitly rationed care: State of the science review. International Journal of Nursing Studies. Elsevier Ltd; 2015;52:1121–37.

23. Bekker M, Coetzee SK, Klopper HC, Ellis SM. Non-nursing tasks, nursing tasks left undone and job satisfaction among professional nurses in South African hospitals. Journal of Nursing Management. 2015;23:1115–25.

24. Lake ET, de Cordova PB, Barton S, Singh S, Agosto PD, Ely B, et al. Missed Nursing Care in Pediatrics. Hosp. Pediatr. American Academy of Pediatrics; 2017;7:378–84.

25. Tubbs-Cooley HL, Pickler RH, Younger JB, Mark B a. A descriptive study of nurse-reported missed care in neonatal intensive care units. Journal of Advanced Nursing. 2014;1–12.

26. Murphy GA V, Gathara D, Aluvaala J, Mwachiro J, Abuya N, Ouma P, et al. Nairobi Newborn Study: a protocol for an observational study to estimate the gaps in provision and quality of inpatient newborn care in Nairobi City County, Kenya. BMJ Open. 2016;6:e012448.

27. Murphy GA V., Gathara D, Abuya N, Mwachiro J, Ochola S, Ayisi R, et al. What capacity exists to provide essential inpatient care to small and sick newborns in a high mortality urban setting? - A cross-sectional study in Nairobi City County, Kenya. PLoS One. 2018;13:e0196585.

28. Murphy GA V, Omondi GB, Gathara D, Abuya N, Mwachiro J, Kuria R, et al. Expectations for nursing care in newborn units in Kenya – moving from implicit to explicit standards. BMJ Global Health.

29. Gould JB, Qin C, Marks AR, Chavez G. Neonatal mortality in weekend vs weekday births. JAMA. 2003;289:2958–62.

30. Concha OP, Gallego B, Hillman K, Delaney GP, Coiera E. Do variations in hospital mortality patterns after weekend admission reflect reduced quality of care or different patient cohorts? A population-based study. BMJ Quality and Safety. 2014;23:215–22.

31. Kalisch BJ, Landstrom GL, Hinshaw AS. Missed nursing care: A concept analysis. Journal of Advanced Nursing. 2009;65:1509–17.

32. Leonard KL, Masatu MC. The use of direct clinician observation and vignettes for health services quality evaluation in developing countries. Social Sciences in Medicine. 2005;61:1944–51.

33. Leonard K, Masatu MC. Outpatient process quality evaluation and the Hawthorne Effect. Social Sciences in Medicine. 2006;63:2330–40.

For peer review only - http://bmjopen.bmj.com/site/about/guidelines.xhtml

34. Isono H, Suzuki S, Ogura J, Haruta J, Maeno T. Improving the workflow of nursing assistants at a general hospital in Japan. BMJ open Quality. 2017;6:e000106.

35. Ministry of Health. Kenya. THE KENYA HIV TESTING SERVICES GUIDELINES. Nairobi, Kenya; 2015.

36. Ministry of Health. Division of of Reproductive Health. National Reproductive Health Research Guidelines. Nairobi, Kenya; 2006.

37. Keats EC, Ngugi A, Macharia W, Akseer N, Khaemba EN, Bhatti Z, et al. Progress and priorities for reproductive, maternal, newborn, and child health in Kenya: a Countdown to 2015 country case study. Lancet Global Health. 2017;5:e782–95.

38. Ministry of Health. Task Sharing Policy Guidelines. 2017.

For peer review only

For peer review only - http://bmjopen.bmj.com/site/about/guidelines.xhtml

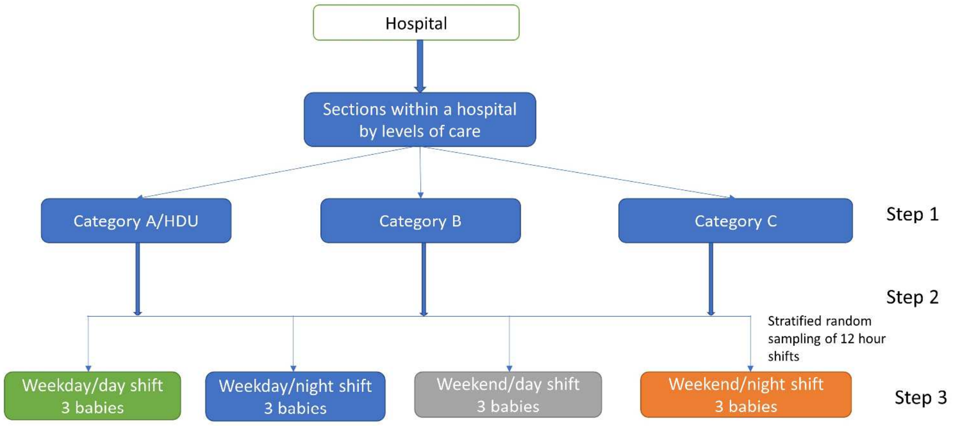

Multi-stage sampling procedure within a hospital for selecting newborns for direct observation

338x190mm (300 x 300 DPI)

For peer review only - http://bmjopen.bmj.com/site/about/guidelines.xhtml

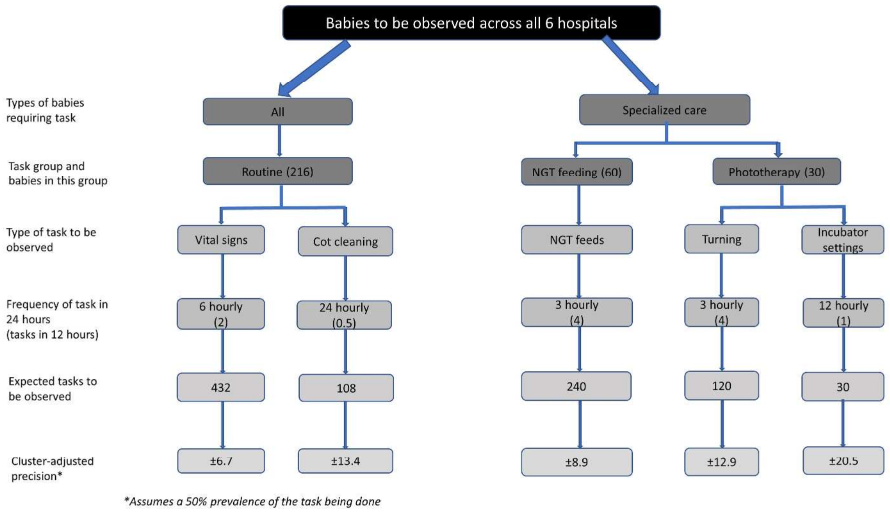

Precision levels for different newborn sub-populations and tasks

338x190mm (300 x 300 DPI)

Category of neonate _ _ Study ID _ _|_ _

Quantifying neonatal nursing care observational checklist

Hospital logistics

1. Start of observation: Date ☐ Time ☐
2. End of observation: Date ☐ Time ☐
3. Shift: Weekday day ☐ Weekday night ☐

   Weekend day ☐ Weekend night ☐
4. Total number of patients in the ward: ☐
5. Total Number of:
   - Admissions ☐
   - Discharges ☐
   - Referrals ☐
   - Death ☐
6. Number of nurses on shift:
   - Straight day shift ☐
   - Morning shift ☐
   - Afternoon shift ☐
   - Night shift ☐
7. Number of other staff providing care other than the nurse:
   - Doctor ☐
   - Clinician ☐
   - Health care assistant ☐
   - Student nurse ☐
   - Student CO ☐
   - Upgrading nurse students ☐

   Others ______________________________

For peer review only

For peer review only - http://bmjopen.bmj.com/site/about/guidelines.xhtml

Bio data

8. Child category: A. (Critical/HDU) ☐
   B. (Acute) ☐
   C. (Stable) ☐
9. Date of admission ☐
10. Age (days/ If day 1 of life give hours) ☐
11. Gender: Male ☐
    Female ☐
12. Admission diagnosis: ______________________________
    ____
13. Current diagnosis: ______________________________
    ____
14. Birth weight (grams) ☐
15. Current weight (grams) ☐ Date taken ☐
16. Type of delivery: SVD ☐ CS ☐ AVD ☐

For peer review only

For peer review only - http://bmjopen.bmj.com/site/about/guidelines.xhtml

Category of neonate _ _ Study ID _ _|_ _

Tasks	Frequency of task	Expected number tasks to be done(12hours/shift)	Tasks_done	Time task done	Comments
**Routine Tasks**					
Handing over		1			
Patient assessments performed each shift		1			
Cleaning of baby		1			
Change of linen		1			
Checking incubator settings		1			
Nurse attends ward round with doctor(s)		1			
Weight check		1			
Elimination care		1			
Discharge planning		1			
Communication/counselling to parent		1			
Hand washing/ Hand rub using sanitizer		1			
Cord care		1			
Checking cannula site		1			
**Regular Tasks**					
Checking vital signs*	4/6 Hourly	3			
Temperature check*	4/6 Hourly	3			
Pulse check*	4/6 Hourly	3			
Respiratory rate check*	4/6 Hourly	3			
SpO2 check*	4/6 Hourly	3			
Turning	4 Hourly	3			
Cup/spoon feeding	3Hourly	4			
Breast feeding	3 Hourly	4			

*Dependend on neonate category

For peer review only - http://bmjopen.bmj.com/site/about/guidelines.xhtml

Tasks	Frequency of task	Expected number tasks to be done(12hours/shift)	Tasks_done				Time task done				Comments
**Critical Tasks**											
**Naso gastric tube feeding**											
**Checking for correct position of tube**	3 Hourly	4									
**Checking gastric aspirate before feeding**	3 Hourly	4									
**Checking actual volume of feeds**	3 Hourly	4									
**Actual feeding**	3 Hourly	4									
**Positioning baby after feeding**	4 Hourly	4									
**Phototherapy**											
**Turning/positioning**	4 Hourly	3									
**Skin assessment**	6 Hourly	2									
**Checking eyes if protected from damage**	6 hourly	2									
**Changing eye pad**	12 Hourly	1									
**Eye care**	12 Hourly	1									
**Oxygen therapy**											
**Checking tube position and nostril-care, damage**	3 Hourly	4									
**Regulating oxygen flow**	12 Hourly	1									
**Intravenous drug administration**											
**Dilutions and checking compatibility**		2									
**Review of treatment sheet**		2									
**Flushing cannula before administering drug**		2									
**Administration of medication**		2									
**Flushing cannula after giving medication**		2									

For peer review only - http://bmjopen.bmj.com/site/about/guidelines.xhtml

Category of neonate _ _ Study ID _ _|_ _

Tasks	Frequency of task	Expected number tasks to be done(12hours/shift)	Tasks_done	Time task done	Comments
**Intravenous Fluid administration**					
Review of treatment sheet		1			
Priming of giving set		1			
Flushing cannula before starting the fluid		1			
Administration and regulating flow rate		1			
**Kangaroo Mother Care**					
Support for KMC		1			
Supervision of mother during KMC		1			

Documentation of tasks

Tasks	Done	Not done	Comments
Neonatal assessment (nursing cardex)			
Planned care (cardex/care plan)			
Vital signs (observation charts/cardex)			
Treatment (treament sheets)			
Ward round comments (cardex)			
Phototherapy (cardex)			
Feeds (feeding chart)			
Oxygen therapy (cardex)			
Health talks/ Communication to parent (cardex)			
Fluids (input output chart)			

For peer review only - http://bmjopen.bmj.com/site/about/guidelines.xhtml

**Turning/position(cardex and/or turning chart)**	☐	☐	
**Alternate weight check**	☐	☐	

**Treatment**			
**Drug name**	**Frequency**	**Times administered in the last 24 hours**	**Comments**

Tasks observed for other babies

**Task**	**Observed**	**Comments**
**Administration of vaccines**		
**Taking venous blood**		
**Taking heel prick**		
**Blood/exchange transfusion**		
**CPAP(setup of machine n tubings)**		
**Resuscitation**		

**Other tasks observed not mentioned above**

For peer review only

BMJ Open

Quantifying nursing care delivered in Kenyan newborn units: Protocol for a cross-sectional direct-observational study

Journal:	*BMJ Open*
Manuscript ID	bmjopen-2018-022020.R2
Article Type:	Protocol
Date Submitted by the Author:	18-Jun-2018
Complete List of Authors:	Gathara, David; Kenya Medical Research Institute/Wellcome Trust Research Programme, Department of Public Health Research<br>Serem, George; Kenya Medical Research Institute/Wellcome Trust Research Programme, Department of Public Health Research<br>Murphy, Georgina ; Kenya Medical Research Institute/Wellcome Trust Research Programme, Department of Public Health Research; Bill and Melinda Gates Foundation<br>Abuya, Nancy; Kenya Medical Research Institute/Wellcome Trust Research Programme, Department of Public Health Research; Nairobi City County, Department of Curative and Preventative Services<br>Kuria, Rose; Kenya Medical Training College, Department of Nursing<br>Tallam, Edna; Nursing Council of Kenya, Department of Registration and Licensing<br>English, Mike; University of Oxford, Nuffield Department of Medicine and Department of Paediatrics; Kenya Medical Research Institute/Wellcome Trust Research Programme, Department of Public Health Research
**Primary Subject Heading**:	Nursing
Secondary Subject Heading:	Health services research, Research methods
Keywords:	Health & safety < HEALTH SERVICES ADMINISTRATION & MANAGEMENT, NEONATOLOGY, STATISTICS & RESEARCH METHODS

SCHOLARONE™
Manuscripts

Quantifying nursing care delivered in Kenyan newborn units: Protocol for a cross-sectional direct-observational study

Investigators and Institutional Affiliations

David Gathara[1*], George Serem[1], Georgina A. V. Murphy [1, 2], Nancy Abuya[1,3], Rose Kuria[4], Edna Tallam[5], Mike English [1,2]

Affiliations

1. Department of Public Health Research, Kenya Medical Research Institute/Wellcome Trust Research Programme, Nairobi, Kenya
2. Nuffield Department of Medicine and Department of Paediatrics, University of Oxford, UK
3. Department of Curative and Preventative services, Nairobi City County, Kenya
4. Department of Nursing, Kenya Medical Training College, Kenya
5. Department of Registration and Licensing, Nursing Council of Kenya

*Corresponding author
Dr David Gathara
P.O Box 43640 00100
Kenya Medical Research Institute/Wellcome Trust Research Programme
Nairobi, Kenya
Email: Dgathara@kemri-wellcome.org
Phone: (+254) 721659321; (+254) 730 162 000

Abstract

Introduction: In many African countries, including Kenya, a major barrier to achieving child survival goals is the slow decline in neonatal mortality that now represents 45% of the under-5-mortality. In newborn care, nurses are the primary care givers in newborn settings and are essential in the delivery of safe and effective care. However, due to high patient workloads and limited resources, nurses may often consciously or unconsciously prioritise the care they provide resulting in some tasks being left undone or partially done (missed care). Missed care has been associated with poor patient outcomes in high income countries. However, missed care, examined by direct observation, has not previously been the subject of research in LMICs.

Methods and analysis: The aim of this study is to quantify essential neonatal nursing care provided to newborns within newborn units. We will undertake a cross-sectional study utilising direct-observational methods within newborn units in six health facilities in Nairobi City County across the public, private-for-profit and private-not-for-profit sectors. A total of 216 newborns will be observed between 1st September 2017 to 30th May 2018. Stratified random sampling will be used to select random 12-hour observation periods while purposive sampling will be used to identify newborns for direct-observation.

For peer review only - http://bmjopen.bmj.com/site/about/guidelines.xhtml

We will report the overall prevalence of care left undone, the common tasks that are left undone and describe any sharing of tasks with people not formally qualified to provide care.

Ethics and dissemination: Ethical approval for this study has been granted by the Kenya Medical Research Institute Scientific and Ethics Review Unit. Written informed consent will be sought from mothers and nurses. Findings from this work will be shared with the participating hospitals, an expert advisory group that comprises of members involved in policy making and more widely to the international community through conferences and peer-reviewed journals.

Strengths and limitations

- The use of direct observational methods to quantifying nursing care delivered or left undone is an approach that has not been previously used in LMICs.
- Different sectors (public, private-for-profit, private-not-for-profit) in health service provision have been included in this study.
- The study provides a 24-hour assessment of neonatal nursing care provision including care provided on weekends and weekdays.
- Our scope is limited by the few number of newborns to be observed within each hospital.
- Despite our efforts to minimize the Hawthrone effect, we cannot rule out the possibility of nurses changing the way they provided care during the observational periods.

Introduction

Despite progress globally, most African countries including Kenya have made insufficient progress in reducing child mortality. In most countries, this can be partly attributed to only slow declines in neonatal mortality. As a consequence, about 45% of mortality for children under-5 years is attributable to neonatal mortality[1]. Of these neonatal deaths, approximately 75% occur in the first 7 days of life and half of these within 24 hours of life[2][3]. A recent review by Bhutta and colleagues indicated that high impact low cost interventions could avert more than 71% of neonatal deaths with 82% of this effect being attributable to facility-based care[4]. However, reports from low income settings highlight that the quality of newborn care in health facilities is often poor[5][6][7]. Therefore, strengthening the quality of facility based care for newborns will be essential in improving newborn outcomes.

Human Resources for Health (HRH) inadequacies is a major factor limiting delivery of quality neonatal services [8]. Globally, the shortage of health workers is estimated currently at over 7 million and by 2035 the deficit is estimated to be 12.9 million[9]. The shortages in the available workforce are worst in developing countries where inequitable distribution of available health workers may compound the problems. In Kenya, Wakaba and colleagues reported that public sector nursing densities ranged between 0.008 to 1.2 per 1000 population across counties[10] compared to an internationally suggested

For peer review only - http://bmjopen.bmj.com/site/about/guidelines.xhtml

minimum health workforce threshold of 2.5/1000 population for doctors, nurses and midwives. In Nairobi County, the nurse densities ranged between 0.21 to 0.40 per 1000 population[10].

There is little specific exploration of the impact of nursing workforce shortfalls on inpatient care in Low and Middle-Income Countries (LMIC). Yet to improve quality of care it is essential that we understand who delivers care (what tasks are done by whom), how care is delivered (how are tasks performed) and critically analyse what tasks are left undone. In most health systems, nurses are gatekeepers of the health care being delivered. They are vested with the responsibility of delivering interventions prescribed by other providers (doctors, nutritionists etc) in addition to providing nurse initiated interventions[11]. As a consequence, few interventions reach the patient without the involvement of the nurse. Yet the few existing evaluations of the quality of care provided to newborns in LMIC have focused on the more medical aspects of care[6,12,13][14]. In LMIC facilities, large patient workloads, insufficient staff and resources, urgent patient situations, and unexpected rises in patient volume and/or acuity on the unit (among other factors) might result in all facets of nursing care being delayed or neglected. This phenomenon has been described as 'implicit rationing'[15], 'missed care'[16], or 'unmet nursing care needs'[17], 'care left undone'[18], or 'task incompletion'[19]. Hereafter, we use the term missed care to encompass all of these terms. Such missed care may have a particularly devastating impact on outcomes in newborn units where nurses are the primary care givers to this highly dependent group.

Justifying a focus on missed nursing care several studies have reported associations between missed care and patient outcomes[20,21][22]. Although there is a growing literature on missed care the majority is from high resource settings, with only one study in South Africa[23] providing an early formal attempt to quantify the extent of the problem in this middle income country. Furthermore, almost all the literature on missed care is based on nurse surveys with only two focusing on newborn care provision[24,25]. Although, nurse surveys on missed care have proven useful, there is a call to undertake more research with a special focus on objective observational methods as no studies of this type were identified in a recent systematic review [22]. The proposed study aims to characterise the care delivered (tasks done or left undone and who does these tasks) to newborns receiving care within newborn units in Nairobi, Kenya by making direct observations of care being provided. This will provide in-depth and objective insights on missed care with particular reference to locally agreed standards for nurses providing neonatal care.

Methods and analysis

This is a cross-sectional study that will involve direct observation of care provided to individual newborns with the aim of describing the essential neonatal nursing care given or missed within newborn units. It will be undertaken in 6 hospitals in Nairobi City County, Kenya, in the period 1st September 2017 to 30th May 2018.

Study site

The proposed research work will be undertaken as part of a broader set of work being conducted in collaboration with Nairobi City County. This collaboration includes work to characterise all the facilities providing inpatient newborn care 24 hours, 7 days a week (hereafter referred to as 24/7) in Nairobi [26],

For peer review only - http://bmjopen.bmj.com/site/about/guidelines.xhtml

quality of clinical care provided newborns[27], and ethnographic work to understand the wider context and practice of neonatal nursing. Based on findings from the broader study, Nairobi county has 34 health facilities providing 24/7 inpatient newborn care, of these, two small health facilities declined to take part in prior quality of care surveys and were estimated to have less than 50 neonatal admissions each per year[27]. Excluding the military health facility with restrictive admission policy, the remaining 31 health facilities that form the population for this study provide 99% of all inpatient neonatal care.

This current study will focus on primary referral (secondary care) facilities that have more than 100 neonatal admissions annually. As such, the two health facilities that declined to participate in prior quality of care surveys do not meet this criterion and are unlikely to introduce selection bias. Thirteen facilities meet these criteria and together provide care to over 96% of the sick-newborn population accessing care within Nairobi County[27]. These 13 facilities will be stratified by workload (newborn admissions per year <=500 low; >500 high) and six health facilities purposefully selected to ensure representation of two hospitals in each of the public, private-not-for profit and private-for-profit sectors, with one high and one low workload facility in each sector. Purposeful selection will be used as it is important in this sensitive and innovative work to have the strong support of the hospital administration. This initial work will therefore help illustrate the nature and magnitude of the challenge of missed care but not make claims to provide a statistically representative picture of missed care which would be challenging given the great diversity of facilities found in Nairobi, with some health facilities having as low as 10 neonatal admissions per year[27].

Study populations

All newborns admitted within the newborn unit in the 6 selected health facilities over the period of the study in each facility will form the potential study population. However, newborns meeting the following exclusion criteria will not be observed: i) newborns requiring specialised treatment to whom the draft minimum nursing standards for neonatal care[28] may not be applicable, for instance newborns with gross malformations or those receiving post-operative care, ii) newborns who are critically ill and at risk of death within a 12-hour observation period as defined by the clinician in charge of the newborn unit for whom observation might cause distress to families, iii) newborns for whom guardians do not provide consent, and iv) newborns receiving care from nurses or guardians who decline to be observed in the care provision process.

Sampling procedures

To describe the care being provided to newborns admitted in newborn units and the spectrum of inpatient services they receive, we are aiming to sample time in 12-hour shifts randomly using the steps described in figure 1.

Figure 1: Steps for the sampling procedure

Step 1

Care within the newborn unit can be organised in a way that babies requiring different levels of care are in different sections/rooms. Intensive care employing invasive mechanical ventilation is not available

For peer review only - http://bmjopen.bmj.com/site/about/guidelines.xhtml

outside tertiary hospitals in Kenya and thus definitions for different levels of newborn care provided by County Hospitals were adopted. These are taken from draft nursing standards for neonatal care that categorise newborn unit sections (and thus babies) in the following way[28]. **Category A (high dependency unit, HDU)**: Babies on oxygen/CPAP and intravenous fluids who are often acutely ill and unstable and require the closest monitoring. **Category B**: Babies who have stabilized but may still be ill and receiving, for example, assisted feeding (nasal-gastric feeds), intravenous drugs; or being observed for convulsions or apnoea. **Category C**: Babies who are quite stable who should be receiving KMC, or stable abandoned babies, or recovering babies requiring completion of treatment such as the last doses of antibiotics or transitioning to oral feeding. Our primary sampling strategy will be based on identifying these organisational sub-sections (Category A; B; C rooms or incubator/cot spaces) in each facility. Where facilities have no clear organisational demarcation into Category A, B and C sub-sections, we will adapt the observations to suit the organisation of care in each facility. However, we will endeavour to identify and select, for observation, newborns with varying degrees of illness severity who would be classified as meeting criteria for Category A, B or C in such settings.

Step 2

In each hospital, stratified-random sampling will be used to generate a random sample of twelve shifts/time blocks of 12 hours (144 observation hours per hospital) from within a three week period stratified by disease severity (category A, B, C), weekdays and weekends as well as night and day shifts as care has been shown to vary across these periods[29,30]. The 12 shifts will be divided equally across the newborn unit sections within a hospital where there is more than one section. Where all babies are cared for in one room without clear sub-sections all observation periods will be conducted in this same setting with efforts to observe babies requiring different levels of care.

Step 3

We feel that it is logistically feasible for one observer to make direct observations of three babies located in adjacent cots in the same ward area at one time. Therefore, for each shift and section, three babies who meet the inclusion criteria will be purposefully selected to ensure babies are in one ward area and within close proximity to allow direct observations. In smaller units where the number of babies per section may be less than three, we will observe all the babies available in the section and classify them as eligible for care in category A, B or C.

Sample size

This is an exploratory cross-sectional study and as such, we illustrate the precision with which we can report proportions of tasks done (or not done) assuming different sized denominators. The size of the denominator is related to the recommended frequency that tasks should be performed (see figure 2). To estimate the precision of reporting we have used a sample estimation approach for cluster designs and assuming a design effect of 2 to adjust for clustering of observed tasks around individual newborns within hospitals.

For peer review only - http://bmjopen.bmj.com/site/about/guidelines.xhtml

At most we expect to recruit 216 newborns across six hospitals (36 newborns per hospital with three babies for each of the 12 shifts/time blocks). For tasks that would be conducted with an expected frequency of once in 12 hours (e.g. intravenous penicillin administration) we might therefore observe 216 task opportunities. Taking a (statistically) conservative assumption that on 50% of expected occasions the task is observed to be done then we could report the proportion of such a task being done with a precision of ± 13.4%. Similarly, if a task should be conducted every six hours then the denominator would be 432 expected tasks and if half were observed the precision of this estimate would be ± 6.7%.

Not all babies observed will require all tasks. For example, some babies may not be receiving intravenous drugs. This will reduce the effective size of the denominator and reduce the precision we can report. Similarly, if babies have to leave the area of observation (for example they are moved to a new ward area or are sent for X-ray etc) this may reduce the number of expected tasks that can be observed. In addition, failure to recruit three babies at each 12-hour shift at each facility may also reduce the number of tasks observed. We illustrate the effect this has on the precision of reported estimates in figure 2, which illustrates that with as few as 30 expected tasks, reporting a precision of ± 20.5% is possible.

As this work is a first of its kind (globally as far as we are aware) we feel that providing estimates of the proportion of tasks done/left undone with a precision of approximately ± 20% will be sufficient to provide valuable insights into the challenges faced by nurses in providing newborn care.

Figure 2: Precision levels for different newborn sub-populations and tasks

Procedures

For each newborn being observed, the diagnosis and disease severity information will be collected from medical records as this informs the expected number of tasks. At the beginning of every shift for which observations will be made, the total number of nursing tasks expected to be delivered will be determined with reference to the medical and nursing records (for disease severity and specific interventions like phototherapy) and general aspects of care each baby should receive. For each of the newborns selected to participate in the study, we will make direct observations on how often certain routine nursing tasks (listed in table 1) are undertaken in a 12-hour shift (7am -7pm or 7pm to 7am) using an observation checklist. Observations will be stopped if a baby is transferred out of a section, changes condition and becomes critically ill (requires specialised treatment which the minimum draft nursing standards for neonatal care do not apply) or discharged, however, we will use the data collected up to the point of exit and the number of observation hours will be documented. If the babies' condition changes and their category changes but they remain in the same observation area, we will document this change and revise the expected number of tasks. In both instances the effective denominator for expected nursing tasks will be changed as required.

The observer will be stationed in a ward area where they do not obstruct care provision but can observe the care being provided to the newborns selected for observation. Because most of the documentation activities happen at the nurses' desk/station, all tasks related to documentation cannot be observed

For peer review only - http://bmjopen.bmj.com/site/about/guidelines.xhtml

from the cot-side. The documented tasks for the babies under observation will be evaluated at the end of each 12-hour shift for which observations will be made. We acknowledge that 12 hours is a long period and the observer's efficiency for making observations might reduce as time progresses within an observation time block and study period. We will factor in rest periods in the 12-hour shifts that coincide with when nurses take their breaks, for instance, during tea and lunch breaks, periods which we anticipated limited or no tasks will be undertaken. Further, to allow enough rest between observation time blocks, we will aim to have a maximum of three, 12-hour observation periods per week per observer with at least a 24-hour rest period between observations. The 12-hour periods were selected because they span nursing shift change overs that allowed documentation of care round-the-clock and made random selection of time blocks more feasible.

The observation checklist (supplementary file 1) is based on nursing standards produced after two expert group meetings held in November 2015 and July 2016 at the KEMRI Wellcome Trust Research Programme offices. The nurse stakeholder group comprised expert nurses who are in the teaching profession or senior practitioners and including the acting Chief Nursing Officer for Kenya. They defined a minimum standard for performing nursing tasks on newborn units[28]. A sub-set of tasks identified as critical (listed in table 1) by the nurse stakeholder group was explicitly marked as to only be performed by nurses and not by other personnel due to the skills required when they are delivered. Where necessary tasks are broken down into manageable observable task components to facilitate observation. For instance, naso-gastric feeding is broken down into insertion of the NGT (as required), checking for gastric aspirate before feeding, counter checking feed volumes to be given with prescription, actual feeding and charting the feeds given. The purpose of observation is not to assess how well any particular aspect of a task is done (eg. the care taken in administering naso-gastric feeds) but simply to determine if the task (or task component) was (or was not) done at all.

Prior to the start of the study, the observation checklist (supplementary file 1) will be extensively piloted over a period of six weeks to determine the quantity and quality of data that can be reasonably gathered and will be adapted as needed. The tool will be piloted in one public health facility that will not be used as a study site in the final study by the research assistant who will subsequently be responsible for training the data clerks. During piloting, we will observe care provision in each of the nursing 12-hour time blocks to explore what tasks can be observed, what number of newborns will be logistically feasible to observe, the different nursing routines in the different shifts and the documents used for reporting on nursing activities.

Table 1: Routine and critical tasks for observation

Routine tasks	Critical tasks
Patient assessment at the beginning of each shift<br>Cleaning of the baby<br>Changing baby's linen<br>Changing the baby's position<br>Checking incubator settings<br>Ward round attendance and active note	Naso-gastric feeding<br>• Insertion of the NGT<br>• Testing whether it is in the correct position<br>• Checking for gastric aspirate before feeding<br>• Preparation of feeds and counter checking feed volumes<br>• Actual feeding and charting the feeds

For peer review only - http://bmjopen.bmj.com/site/about/guidelines.xhtml

taking<br>Weighing<br>Elimination care<br>Communication/counselling parents<br>Cord care<br>Vital signs measurement<br>• Pulse rate<br>• Temperature<br>• Respiratory rate<br>• Oxygen saturation<br>Documentation<br>• Updating the nursing cardex<br>• Discharge and admission registration	Intravenous drug/fluid administration<br>• Reviewing treatment sheet<br>• Checking cannula sites – care<br>• Regulating flow<br>• Input/output charting for fluids<br>• Document treatments given<br>Oxygen therapy<br>• Fixing of oxygen/nasal prongs<br>• Checking tube position and nostril –care, damage<br>• Initiating and regulating oxygen flow<br>• Documenting oxygen treatment<br>Photo therapy<br>• Baby positioning<br>• Placing/checking eye pad is in place<br>• Checking eyes for damage<br>• Checking and monitoring phototherapy settings<br>• Documenting of phototherapy<br>Support for KMC

For purposes of this study, we adopted the definition of missed care reported in wider literature[22,31], and was therefore defined as care that the nursing advisory group regard as necessary (primarily essential neonatal nursing tasks) as part of routine newborn care that are left undone or are delivered by any person other than the nurse or a qualified health care provider (nutritionist, doctor, clinical officer etc). However, tasks that will be done by a senior student nurse under direct observation/supervision by the nurse will be considered as done and documented as not done if no supervision is observed. For example, tasks done by a senior student nurse who is being supervised to conduct naso-gastric feeding by a qualified nurse in the room at the time and focused on the supervision will be regarded as done. If the same student does the task while the nurse supervising is in a different part of the ward it will be regarded as not done (with a record of who completed the task made). Additional data to be collected related to each block of observation time will include data on the nurse-patient ratio for the ward as a whole, what additional staff are present within the unit e.g. nursing students, support staff etc. and patient workloads within the different sections of the ward.

Practical considerations for undertaking direct observations

Observations will be made by a person familiar with the hospital environment, equipment, processes (like ward rounds) and language but who is not a nurse or clinician (doctor/clinical officer). Given the potential sensitivity of this form of observation, it is important that this person is considered a professional rather than an 'outsider' (who might not be bound by professional codes of confidentiality). Having an observer who has an understanding of the setting but who is not a clinician or nurse may also overcome problems of the observer making judgements about what is being observed based on their own standards of practice or being influenced as they make observations by

For peer review only - http://bmjopen.bmj.com/site/about/guidelines.xhtml

professional allegiances (eg. a nurse observer may not wish to record that a task is left undone). There might also be ethical challenges for an observer with a clinical/nurse background who might feel obliged to intervene in the provision of care or become co-opted to complete tasks. An observer who is not licensed to offer the form of interventions/care being given to babies will still however be able to report to health care providers within the unit in the event they identify gaps or situations in which newborns are put at risk. For instance, if they see a newborn convulsing or vomiting, they will alert the nurse. Suitable backgrounds for observers might include nutritionists, health records officers, ward based clerical workers, or laboratory or pharmacy staff with at least two-year's experience within hospital settings. In this study nutritionists will be recruited to undertake the observations.

We will recruit six observers who meet the above criterion. The observers will be trained for a period of two weeks by one of the study personnel who has a nursing background and has two years' experience providing care to newborns within a newborn unit. Standard operating procedures (SOPs) will be developed and will serve as a guide for the observational work. During the training period, the observers and the trainer will do observations and comparisons will be made. Any differences will be discussed and where necessary the SOPs will be revised to improve on clarity. Supervision during the data collection period will be undertaken by study staff with a nursing background with routine weekly reviews of the observation checklists for completion and consistency.

To reduce the Hawthorne effect where nurses might change the way they provide care when they are being observed, the observer will spend at least one week in the health facility before starting the observational work. This will also allow him/her to familiarise themselves with the environment, to explain the study to staff and parents and for the staff within the newborn unit to get used to them. In addition, the observer will make it clear that the observations are not an assessment but a means of understanding what care is possible to provide given existing resource constraints. Formal study observations will then begin in the period after the one-week familiarisation period. This approach is supported by evidence that health care providers change their practice slightly when the observations start but these changes are short-lived and quickly dissipate with health care providers soon reverting to their previous practice[32,33]. In addition, the 12-hour observation periods are randomly generated and the hospitals will not be aware of the day, shift or category of babies the observer would next come to do observations for.

Practical and public involvement

Patients and the public were not involved in the development of the research question or outcome measures. However, as part of the broader set of work within which this study is embedded, there is ongoing work to understand patient experiences focusing on experiences of mothers with newborns admitted within the newborn unit. We hope findings from our study will compliment those from the patient experiences work and will provide insights on aspects of care that are important to mothers but also inform the design of interventions to improve care.

Data Management and analysis

For peer review only - http://bmjopen.bmj.com/site/about/guidelines.xhtml

Data will be collected on paper-based observation checklists, one for each baby that is the focus of observation. No participant identifiable data will be collected and data collection instruments will be identified only by a unique study ID allocated to each baby. The place, date and time of the shift will also be anonymised by using only specific codes for observation shifts at data entry. Data on the paper-based observation checklists will be checked for completeness by a supervisor at the end of each day. The checklists will be double-entered into a custom-made database using Research Electronic Data Capture (REDCap) with inbuilt range and consistency checks. The entered data will be checked at the end of each day using pre-coded scripts for entry errors and completeness. Data will be exported for cleaning and analyses in Stata version 13 (Stata Corporation, Texas, USA).

Descriptive analysis will be undertaken on the pooled data across hospitals to determine the overall prevalence of care left undone and the common tasks that are left undone. We will also report the average number of tasks left undone per newborn and the common tasks left undone per newborn. Secondary analysis will be undertaken to explore variations in care done (or left undone) by the various sub-categories that will include: sector, nursing shift, nurse-patient ratios and category of the baby (disease severity). Missing or incomplete data will be coded as a category and where necessary presented as such. When reporting on effective number of tasks done (or left undone), missing/incomplete data will be excluded from the effective denominator the task would have contributed to and hence avoid spurious inflation of the denominator

Ethical considerations

The focus of our direct observations of care is what happens to newborns and we will not record any names or other identifying features of people who may be providing care to these newborns. We will only report pooled results stratified by sector, nurse-patient staffing ratios and category/severity of disease so as to preserve the confidentiality of the health facilities.

During the one-week familiarisation period, we will seek written individual informed consent from all nurses who will be providing care within the newborn unit. Additional consent forms and study briefs will be left in the ward to allow nurses not available during this introductory period but who provide care in the newborn unit to review and indicate their willingness to participate. During an observation shift these nurses will be asked for individual informed consent before the start of the observations. Additionally, at the beginning of every shift for which direct observations will be made, group verbal informed consent will be sought from nurses after an explanation of the study has been made (this is in addition to the one-week familiarisation during which the study will be explained). We will also provide printed study briefs targeting health care providers explaining the study but also indicating that they are free to decline from being observed as an approach to on-going consent.

Written informed consent will be sought from the mothers of babies considered for direct observations at the start of each observation period. In Kenya, pregnant adolescents between ages (15 – 17 years) are considered ‘emancipated minors’ and their written informed consent will be obtained[34–36]. The start of the direct observation shift (7am or 7pm) is just before when mothers are in the newborn unit for the 3-hourly feeding session at 6am or 6pm, as such mothers will be approached during this period. In instances, where mothers indicate more time is required to make a decision or to consult they will be allowed to do so and another mother whose baby meets the criteria for observation will be approached.

For peer review only - http://bmjopen.bmj.com/site/about/guidelines.xhtml

In cases where nurses or parents decline consent, babies under their care will not be considered for direct observation. It will be made clear that at any stage nurses or parents can withdraw consent/permission for observation, temporarily or for the rest of a shift, without explanation and with no penalty. Observations will only be undertaken where both the mother and nurses have provided consent to the study respectively.

Ethical approval to conduct this study in all hospitals has been granted by the Kenya Medical Research Institute (KEMRI) Scientific and Ethics Review Unit (Approval No. KEMRI/SERU/CGMR-C/065/3404) while permission to undertake the study in the respective hospitals was sought from each of the hospital's administrative offices.

Dissemination of findings

An expert advisory group, including partners from the Ministry of Health, Nursing Council of Kenya, Nairobi City County, Kenya Medical Training College, Kenyatta University, and hospitals providing inpatient neonatal services has been involved in the development of the standards of nursing care being used to understand missed care in this study. This expert group has provided support for the study and is in itself a key consumer of the results as these members are directly involved in policy making and training. This group will also provide a key channel for wider dissemination of the findings.

Highlighting the extent and magnitude of care left undone will provide important insights on the nursing care available to newborns admitted within newborn units, highlight human resource issues warranting attention and will likely influence recommendations on staffing norms and how care is organised and delivered in newborn units. Further, findings from this work will help guide the design of approaches and interventions for improving facility based care for this highly vulnerable population.

At the end of the study, findings will be provided to and discussed with participating hospitals and other relevant stakeholders. More widely, the international scientific community will be targeted via publications in peer reviewed journals as well as conferences.

Global public health relevance

Facility based care has been cited as one key approach to reduce neonatal mortality if evidence-based, high impact low cost interventions are appropriately delivered at high coverage[4]. Globally, improving quality of newborn care provided by facilities might save 600 000 small and ill neonates annually[4] while data from Kenya suggest that by 2030, 6000 newborn lives could be saved through the provision of childbirth and newborn care intervention packages alone[37].

The Kenyan government is promoting facility based delivery by making maternity care free for women. This has resulted in an increase in utilisation of maternal and newborn health services, potentially increasing the number of newborns accessing care in health facilities. At the same time significant challenges exist in nurse staffing and availability of wider resources[27] and this may limit any impact of increased access to care. The extent of missed nursing care in newborn units in LMIC and how it impacts on quality of care delivered has not previously been described. This is despite care in the newborn unit being heavily dependent on nurses. By characterising care left undone, we will identify important

For peer review only - http://bmjopen.bmj.com/site/about/guidelines.xhtml

potential gaps in care delivered within newborn units that can inform discussions on how best to address these gaps and improve quality of care in Kenyan hospitals.

This study will be the first attempt, of which we are aware, to develop and apply direct observational missed care tools to understanding neonatal nursing care provision in LMIC. In fact, a recent review by Jones and colleagues identified that only questionnaires have previously been used to quantify missed care and these might be limited by reporting bias[22]. However, we acknowledge that direct observational methods have limitations on the number and actual tasks that can be observed, might be influenced by observer bias and are at risk of Hawthorne effect. As such, the tasks in our observation checklist (supplementary file 1)_are limited to essential neonatal nursing tasks provided at the bedside that can be observed while those linked to documentation will involve review of medical records at the nurses' desk since documentation of care is done at the nurses' desk and not at the cot-side. Moreover, observing the documentation of a task or reviewing records for evidence of documentation is likely to provide similar results. An alternative method for data collection that we considered was the use of video in the newborn unit with later evaluation of what is done (or not done). However, informal discussions indicated this might be controversial at this stage due to the ethical and medico-legal issues that might emanate from this approach and the administrative approvals required for video recording in hospitals, nonetheless this is a potential area for future research. The selection of health facilities was purposive, as support by the hospital administration was important due to the nature of data collection and the sensitivity associated with direct observation of care. As such, we cannot rule out selection bias as facilities providing better care might have been more likely to agree to partake in the study. However, this is the first such study using direct observational methods to quantify missed care and in a LMIC and therefore the findings will still be important. We anticipate that this study will, therefore, be of global interest and provide an opportunity for application of the methods developed to other neonatal settings or in other disciplines to better understand nursing care provision and quality gaps. Further, findings of this work will be important in contributing to thinking on nurse staffing norms and how care is organised and delivered in LMIC newborn units. This will be crucial in influencing longer term policy on human resource planning. Most directly the findings will feed into the 'Kenya Task Sharing Policy and Guidelines for Health Care Services'[38], an initiative being led by Ministry of Health alongside other stakeholders as one of the ways of tackling health workforce shortages.

Authors' contributions

DG, ME and GAVM designed the study with contributions from RK and ET. DG, NA, and GS were responsible for the coordination and supervision of data collection. DG wrote the study protocol with substantial and critical input from all co-authors. All authors read and approved the final version of the manuscript.

Funding

This work was supported by the Health Systems Research Initiative joint grant provided by the Department for International Development, UK (DFID), Economic and Social Research Council (ESRC), Medical Research Council (MRC) and Wellcome Trust, grant number MR/M015386/1. ME is supported by a Wellcome Trust Senior Research Fellowship (#097170).

For peer review only - http://bmjopen.bmj.com/site/about/guidelines.xhtml

Competing interests

None

Figure legends

Figure 1: Steps for the sampling procedure

Multi-stage sampling procedure within a hospital for selecting newborns for direct observation

Figure 2: Precision levels for different newborn sub-populations and tasks

Estimated levels of precision for the different newborn sub-populations and tasks observed that the study will report since not all newborns observed will require all tasks.

For peer review only

For peer review only - http://bmjopen.bmj.com/site/about/guidelines.xhtml

1. References

1. World Health Organization. Countdown to 2015: A Decade of Tracking Progress for Maternal, Newborn and Child Survival. Geneva; 2015.

2. Lawn JE, Cousens S, Zupan J. 4 million neonatal deaths : When ? Where ? Why ? Lancet. 2005;365:9–18.

3. Belizán JM, McClure EM, Goudar SS, Pasha O, Esamai F, Patel A, et al. Neonatal death in low- to middle-income countries: a global network study. American Journal of Perinatology. 2012;29:649–56.

4. Bhutta ZA, Das JK, Bahl R, Lawn JE, Salam RA, Paul VK, et al. Can available interventions end preventable deaths in mothers, newborn babies, and stillbirths, and at what cost? Lancet (London, England). Elsevier; 2014;384:347–70.

5. Vesel L, Manu A, Lohela TJ, Gabrysch S, Okyere E, Ten Asbroek AHA, et al. Quality of newborn care: a health facility assessment in rural Ghana using survey, vignette and surveillance data. BMJ Open. 2013;3.

6. Mbwele B, Reddy E, Reyburn H. A rapid assessment of the quality of neonatal healthcare in Kilimanjaro region, northeast Tanzania. BMC Pediatrics. 2012;12:182.

7. Opondo C, Ntoburi S, Wagai J, Wafula J, Wasunna A, Were F, et al. Are hospitals prepared to support newborn survival? - An evaluation of eight first-referral level hospitals in Kenya. Tropical Medicine and International Health. 2009;14:1165–72.

8. Dickson KE, Simen-Kapeu A, Kinney M V., Huicho L, Vesel L, Lackritz E, et al. Every Newborn: Health-systems bottlenecks and strategies to accelerate scale-up in countries. Lancet. 2014;384:438–54.

9. Campbell J, Dussault G, Buchan J, Pozo-Martin F, Arias MG, Leone C, et al. A universal Truth: No Health without a Workforce. World Health Organization, Geneva; 2013.

10. Wakaba M, Mbindyo P, Ochieng J, Kiriinya R, Todd J, Waudo A, et al. The public sector nursing workforce in Kenya: a county-level analysis. Human Resources for Health. 2014;12:6.

11. All-Party Parliamentary Group on Global Health. Triple Impact: How developing nursing will improve health, promote gender equality and support economic growth. 2016.

12. Reyburn H, Mwakasungula E, Chonya S, Mtei F, Bygbjerg I, Poulsen A, et al. Clinical assessment and treatment in paediatric wards in the north-east of the United Republic of Tanzania. Bull World Health. Organ. 2008;86:132–9.

13. Gathara D, Opiyo N, Wagai J, Ntoburi S, Ayieko P, Opondo C, et al. Quality of hospital care for sick newborns and severely malnourished children in Kenya: A two-year descriptive study in 8 hospitals. BMC Health Services Research. 2011. p. 307.

14. Aluvaala J, Nyamai R, Were F, Wasunna A, Kosgei R, Karumbi J, et al. Assessment of neonatal care in clinical training facilities in Kenya. Archives of Disease in Childhood. 2014;2014-306423-.

15. Schubert M, Glass TR, Clarke SP, Schaffert-Witvliet B, De Geest S. Validation of the Basel Extent of Rationing of Nursing Care instrument. Nursing Research. 2007;56:416–24.

16. Kalisch BJ. Missed nursing care: a qualitative study. Journal of Nursing Care Quality. 2006;21:306-13-5.

17. Lucero RJ, Lake ET, Aiken LH. Variations in nursing care quality across hospitals. Journal of Advanced

For peer review only - http://bmjopen.bmj.com/site/about/guidelines.xhtml

Nursing. 2009;65:2299–310.

18. Ausserhofer D, Zander B, Busse R, Schubert M, De Geest S, Rafferty AM, et al. Prevalence, patterns and predictors of nursing care left undone in European hospitals: results from the multicountry cross-sectional RN4CAST study. BMJ Quality and Safety. 2014;23:126–35.

19. Al-Kandari F, Thomas D. Factors contributing to nursing task incompletion as perceived by nurses working in Kuwait general hospitals. Journal of Clinical Nursing. 2009;18:3430–40.

20. Papastavrou E, Andreou P, Efstathiou G. Rationing of nursing care and nurse-patient outcomes: a systematic review of quantitative studies. International Journal of Health Planning and Management. 2014;29:3–25.

21. Kalisch BJ, Tschannen D, Lee H. Missed Nursing Care, Staffing, and Patient Falls. Journal of Nursing Care Quality. 2011;27:6–12.

22. Jones TL, Hamilton P, Murry N. Unfinished nursing care, missed care, and implicitly rationed care: State of the science review. International Journal of Nursing Studies. 2015;52:1121–37.

23. Bekker M, Coetzee SK, Klopper HC, Ellis SM. Non-nursing tasks, nursing tasks left undone and job satisfaction among professional nurses in South African hospitals. Journal of Nursing Management. 2015;23:1115–25.

24. Lake ET, de Cordova PB, Barton S, Singh S, Agosto PD, Ely B, et al. Missed Nursing Care in Pediatrics. Hosp. Pediatr. American Academy of Pediatrics; 2017;7:378–84.

25. Tubbs-Cooley HL, Pickler RH, Younger JB, Mark B a. A descriptive study of nurse-reported missed care in neonatal intensive care units. Journal of Advanced Nursing. 2014;1–12.

26. Murphy GA V, Gathara D, Aluvaala J, Mwachiro J, Abuya N, Ouma P, et al. Nairobi Newborn Study: a protocol for an observational study to estimate the gaps in provision and quality of inpatient newborn care in Nairobi City County, Kenya. BMJ Open. 2016;6:e012448.

27. Murphy GA V., Gathara D, Abuya N, Mwachiro J, Ochola S, Ayisi R, et al. What capacity exists to provide essential inpatient care to small and sick newborns in a high mortality urban setting? - A cross-sectional study in Nairobi City County, Kenya. PLoS One. 2018;13:e0196585.

28. Murphy GA V, Omondi GB, Gathara D, Abuya N, Mwachiro J, Kuria R, et al. Expectations for nursing care in newborn units in Kenya – moving from implicit to explicit standards. BMJ Global Health.

29. Gould JB, Qin C, Marks AR, Chavez G. Neonatal mortality in weekend vs weekday births. JAMA. 2003;289:2958–62.

30. Concha OP, Gallego B, Hillman K, Delaney GP, Coiera E. Do variations in hospital mortality patterns after weekend admission reflect reduced quality of care or different patient cohorts? A population-based study. BMJ Quality and Safety. 2014;23:215–22.

31. Kalisch BJ, Landstrom GL, Hinshaw AS. Missed nursing care: A concept analysis. Journal of Advanced Nursing. 2009;65:1509–17.

32. Leonard KL, Masatu MC. The use of direct clinician observation and vignettes for health services quality evaluation in developing countries. Social Sciences in Medicine. 2005;61:1944–51.

33. Leonard K, Masatu MC. Outpatient process quality evaluation and the Hawthorne Effect. Social

For peer review only - http://bmjopen.bmj.com/site/about/guidelines.xhtml

Sciences in Medicine. 2006;63:2330–40.

34. Isono H, Suzuki S, Ogura J, Haruta J, Maeno T. Improving the workflow of nursing assistants at a general hospital in Japan. BMJ open Quality. 2017;6:e000106.

35. Ministry of Health. Kenya. THE KENYA HIV TESTING SERVICES GUIDELINES. Nairobi, Kenya; 2015.

36. Ministry of Health. Division of of Reproductive Health. National Reproductive Health Research Guidelines. Nairobi, Kenya; 2006.

37. Keats EC, Ngugi A, Macharia W, Akseer N, Khaemba EN, Bhatti Z, et al. Progress and priorities for reproductive, maternal, newborn, and child health in Kenya: a Countdown to 2015 country case study. Lancet Global Health. 2017;5:e782–95.

38. Ministry of Health. Task Sharing Policy Guidelines. 2017.

For peer review only

For peer review only - http://bmjopen.bmj.com/site/about/guidelines.xhtml

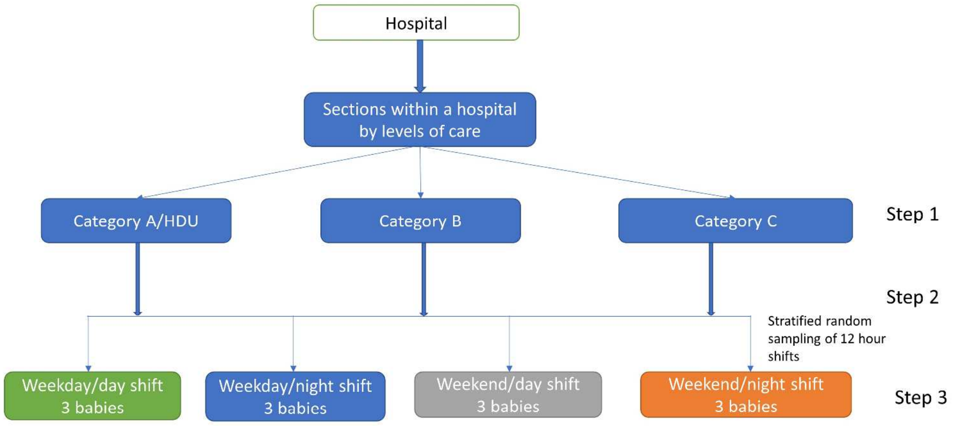

Multi-stage sampling procedure within a hospital for selecting newborns for direct observation

338x190mm (300 x 300 DPI)

For peer review only

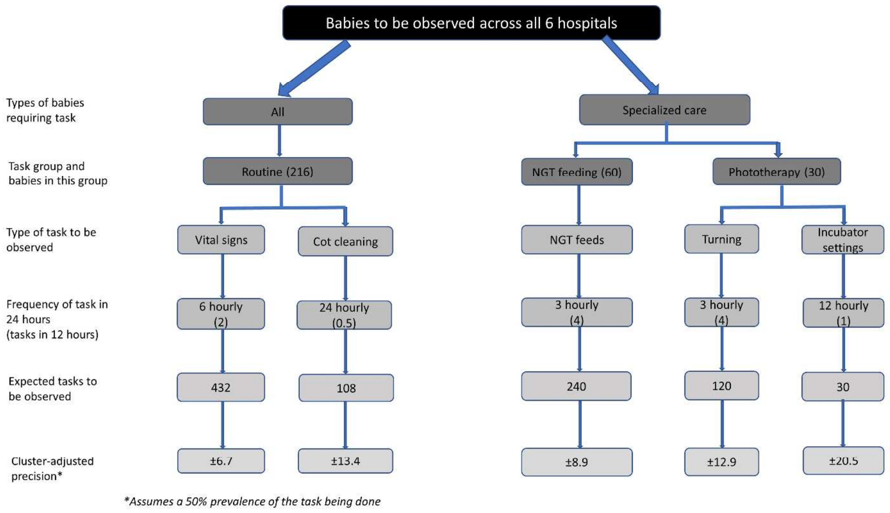

Precision levels for different newborn sub-populations and tasks

338x190mm (300 x 300 DPI)

Category of neonate _ _ Study ID _ _|_ _

Quantifying neonatal nursing care observational checklist

Hospital logistics

1. Start of observation: Date [ ] Time [ ]
2. End of observation: Date [ ] Time [ ]
3. Shift: Weekday day [ ] Weekday night [ ]
   Weekend day [ ] Weekend night [ ]
4. Total number of patients in the ward: [ ]
5. Total Number of: Admissions [ ]
   Discharges [ ]
   Referrals [ ]
   Death [ ]
6. Number of nurses on shift: Straight day shift [ ]
   Morning shift [ ]
   Afternoon shift [ ]
   Night shift [ ]
7. Number of other staff providing care other than the nurse:
   Doctor [ ]
   Clinician [ ]
   Health care assistant [ ]
   Student nurse [ ]
   Student CO [ ]
   Upgrading nurse students [ ]

   Others ______________________________

For peer review only - http://bmjopen.bmj.com/site/about/guidelines.xhtml

Bio data

8. Child category: A. (Critical/HDU) ☐
   B. (Acute) ☐
   C. (Stable) ☐

9. Date of admission ☐

10. Age (days/ If day 1 of life give hours) ☐

11. Gender: Male ☐
    Female ☐

12. Admission diagnosis: ____________________
    ____

13. Current diagnosis: ____________________
    ____

14. Birth weight (grams) ☐

15. Current weight (grams) ☐ Date taken ☐

16. Type of delivery: SVD ☐ CS ☐ AVD ☐

For peer review only

For peer review only - http://bmjopen.bmj.com/site/about/guidelines.xhtml

Category of neonate _ _ Study ID _ _|_ _

Tasks	Frequency of task	Expected number tasks to be done(12hours/shift)	Tasks_done	Time task done	Comments
**Routine Tasks**					
Handing over		1			
Patient assessments performed each shift		1			
Cleaning of baby		1			
Change of linen		1			
Checking incubator settings		1			
Nurse attends ward round with doctor(s)		1			
Weight check		1			
Elimination care		1			
Discharge planning		1			
Communication/counselling to parent		1			
Hand washing/ Hand rub using sanitizer		1			
Cord care		1			
Checking cannula site		1			
**Regular Tasks**					
Checking vital signs*	4/6 Hourly	3			
Temperature check*	4/6 Hourly	3			
Pulse check*	4/6 Hourly	3			
Respiratory rate check*	4/6 Hourly	3			
SpO2 check*	4/6 Hourly	3			
Turning	4 Hourly	3			
Cup/spoon feeding	3Hourly	4			
Breast feeding	3 Hourly	4			
*Dependend on neonate category					

For peer review only - http://bmjopen.bmj.com/site/about/guidelines.xhtml

Category of neonate _ _ | Study ID _ _|_ _

Tasks	Frequency of task	Expected number tasks to be done(12hours/shift)	Tasks_done	Time task done	Comments
**Critical Tasks**					
**Naso gastric tube feeding**					
Checking for correct position of tube	3 Hourly	4			
Checking gastric aspirate before feeding	3 Hourly	4			
Checking actual volume of feeds	3 Hourly	4			
Actual feeding	3 Hourly	4			
Positioning baby after feeding	4 Hourly	4			
**Phototherapy**					
Turning/positioning	4 Hourly	3			
Skin assessment	6 Hourly	2			
Checking eyes if protected from damage	6 hourly	2			
Changing eye pad	12 Hourly	1			
Eye care	12 Hourly	1			
**Oxygen therapy**					
Checking tube position and nostril-care, damage	3 Hourly	4			
Regulating oxygen flow	12 Hourly	1			
**Intravenous drug administration**					
Dilutions and checking compatibility		2			
Review of treatment sheet		2			
Flushing cannula before administering drug		2			
Administration of medication		2			
Flushing cannula after giving medication		2			

For peer review only - http://bmjopen.bmj.com/site/about/guidelines.xhtml

Category of neonate _ _ Study ID _ _|_ _

Tasks	Frequency of task	Expected number tasks to be done(12hours/shift)	Tasks_done	Time task done	Comments
**Intravenous Fluid administration**					
Review of treatment sheet		1	☐	☐	
Priming of giving set		1	☐	☐	
Flushing cannula before starting the fluid		1	☐	☐	
Administration and regulating flow rate		1	☐	☐	
**Kangaroo Mother Care**					
Support for KMC		1	☐	☐	
Supervision of mother during KMC		1	☐	☐	

Documentation of tasks

Tasks	Done	Not done	Comments
Neonatal assessment (nursing cardex)	☐	☐	
Planned care (cardex/care plan)	☐	☐	
Vital signs (observation charts/cardex)	☐	☐	
Treatment (treament sheets)	☐	☐	
Ward round comments (cardex)	☐	☐	
Phototherapy (cardex)	☐	☐	
Feeds (feeding chart)	☐	☐	
Oxygen therapy (cardex)	☐	☐	
Health talks/ Communication to parent (cardex)	☐	☐	
Fluids (input output chart)	☐	☐	

For peer review only - http://bmjopen.bmj.com/site/about/guidelines.xhtml

**Turning/position(cardex and/or turning chart)**	☐	☐	
**Alternate weight check**	☐	☐	

Treatment

**Drug name**	**Frequency**	**Times administered in the last 24 hours**	**Comments**

Tasks observed for other babies

**Task**	**Observed**	**Comments**
**Administration of vaccines**		
**Taking venous blood**		
**Taking heel prick**		
**Blood/exchange transfusion**		
**CPAP(setup of machine n tubings)**		
**Resuscitation**		

**Other tasks observed not mentioned above**

For peer review only - http://bmjopen.bmj.com/site/about/guidelines.xhtml
